# Fair evaluation of global network aligners

**DOI:** 10.1186/s13015-015-0050-8

**Published:** 2015-06-09

**Authors:** Joseph Crawford, Yihan Sun, Tijana Milenković

**Affiliations:** Department of Computer Science and Engineering, Interdisciplinary Center for Network Science and Applications (iCeNSA), ECK Institute for Global Health, University of Notre Dame, Notre Dame, IN 46556 USA; Department of Computer Science and Technology, Tsinghua University, Beijing, 100084 China; Department of Computer Science, Carnegie Mellon University, Pittsburgh, PA 15213-3891 USA

**Keywords:** Protein–protein interaction networks, Network alignment, Network similarity, Across-species protein function prediction

## Abstract

**Background:**

Analogous to genomic sequence alignment, biological network alignment identifies conserved regions between networks of different species. Then, function can be transferred from well- to poorly-annotated species between aligned network regions. Network alignment typically encompasses two algorithmic components: node cost function (NCF), which measures similarities between nodes in different networks, and alignment strategy (AS), which uses these similarities to rapidly identify high-scoring alignments. Different methods use both different NCFs and different ASs. Thus, it is unclear whether the superiority of a method comes from its NCF, its AS, or both. We already showed on state-of-the-art methods, MI-GRAAL and IsoRankN, that combining NCF of one method and AS of another method *can* give a new superior method. Here, we evaluate MI-GRAAL against a newer approach, GHOST, by mixing-and-matching the methods’ NCFs and ASs to potentially further improve alignment quality. While doing so, we approach important questions that have not been asked systematically thus far. First, we ask how much of the NCF information should come from protein sequence data compared to network topology data. Existing methods determine this parameter more-less arbitrarily, which could affect alignment quality. Second, when topological information is used in NCF, we ask how large the size of the neighborhoods of the compared nodes should be. Existing methods assume that the larger the neighborhood size, the better.

**Results:**

Our findings are as follows. MI-GRAAL’s NCF is superior to GHOST’s NCF, while the performance of the methods’ ASs is data-dependent. Thus, for data on which GHOST’s AS is superior to MI-GRAAL’s AS, the combination of MI-GRAAL’s NCF and GHOST’s AS represents a new superior method. Also, which amount of sequence information is used within NCF does not affect alignment quality, while the inclusion of topological information is crucial for producing good alignments. Finally, larger neighborhood sizes are preferred, but often, it is the second largest size that is superior. Using this size instead of the largest one would decrease computational complexity.

**Conclusion:**

Taken together, our results represent general recommendations for a fair evaluation of network alignment methods and in particular of two-stage NCF-AS approaches.

**Electronic supplementary material:**

The online version of this article (doi:10.1186/s13015-015-0050-8) contains supplementary material, which is available to authorized users.

## Background

### Motivation and related work

Analogous to sequence alignment, which finds regions of similarity that are a likely consequence of functional or evolutionary relationships between the sequences, network (or graph) alignment finds regions of topological and functional similarity between networks of different species [1]. Then, functional (e.g., aging-related [2–4]) knowledge can be transferred between species across conserved (aligned) network regions. Thus, just as sequence alignment, network alignment can be used for establishing from biological network data orthologous relationships between different proteins or phylogenetic relationships between different species [5–7]. Also, it can be applied to research problems in other domains, such as semantically matching entities in different ontologies [8], or comparing online social networks with impacts on user privacy [9].

Network alignment can be performed locally and globally. Local network alignment (LNA) aims to optimize similarity between local regions of different networks [10–19]. As such, LNA often leads to many-to-many node mapping between different networks. However, LNA is generally unable to find large conserved subgraphs. Thus, methods for global network alignment (GNA) have been proposed, which aim to optimize global similarity between different networks and can thus find large conserved subgraphs [2, 3, 5–7, 9, 20–31]. Unlike LNA, GNA typically results in one-to-one node mapping between different networks (though some exceptions exist that result in one-to-many or many-to-many node mapping [24, 32]). In this study, we focus on one-to-one GNA due to its recent popularity [2, 3, 31], but all concepts and ideas can also be applied to one-to-many or many-to many GNA, as well as to LNA.

More formally, we define GNA as a one-to-one mapping between nodes of two networks that aligns the networks well with respect to a desired topological or functional criterion. GNA is a computationally hard problem to solve due to the underlying subgraph isomorphism problem [33]. This is an NP-complete problem that asks whether a network exists as an exact subgraph of a larger network. GNA is a more general problem which aims to fit well two networks when one network is not necessarily an exact subgraph of another network. Since GNA is computationally hard, heuristic methods need to be sought. Many (though not all) GNA heuristic algorithms typically achieve an alignment via two algorithmic components: node cost function (NCF) and alignment strategy (AS) [5–7, 25, 26, 30, 34–36]. NCF captures pairwise costs (or equivalently, similarities) of aligning nodes in different networks, and AS uses these costs to identify a good-quality alignment out of all possible alignments with respect to some topological or biological alignment quality measure [2, 3, 5–7, 20, 24–26, 34].

Different existing two-step GNA methods use both different NCFs and ASs, so it is unclear whether the superiority of a method comes from its NCF, AS, or both. For this reason, in our recent study [2, 3], we combined NCFs and ASs of MI-GRAAL [7] and IsoRankN [24], two state-of-the-art methods at the time, as a proof of concept that it is important to fairly evaluate the contribution of each component to alignment quality. In the process, we showed that NCF of MI-GRAAL is superior to that of IsoRankN, and importantly, we proposed the combination of MI-GRAAL’s NCF and IsoRankN’s AS as a new superior method for multiple GNA, i.e., for GNA of more than two networks at a time [2, 3].

In the meanwhile, a new state-of-the-art method has appeared, called GHOST [25]. When recently tested against many other both previous and newer GNA methods, GHOST was described as still “an excellent performer” [31]. Thus, in this study, we aim to understand whether it is GHOST’s NCF or AS (or both) that leads to its good performance, as well as to explore the possibility of further increasing GHOST’s performance by replacing its current NCF with a different, potentially superior NCF. For these reasons, we fairly evaluate MI-GRAAL against GHOST by mixing and matching their NCFs and ASs. We use MI-GRAAL in this study because we already demonstrated the superiority of its NCF, as discussed above [2, 3]. At the same time, we ask several additional important questions regarding the choice of appropriate GNA parameters, which have surprisingly been neglected thus far.

We note that some of the existing one-to-one GNA methods do not belong to this two-stage NCF-AS method category, and clearly, our study might not directly be applicable to such approaches. However, many of the existing one-to-one GNA methods *do* belong to the two-stage category, such as two versions of IsoRank [20, 22], GRAAL [5], H-GRAAL [6], MI-GRAAL [7], and GHOST [25]. It is very likely that many new methods will build on top of these well-established state-of-the-art methods, and thus, our study is of importance for future GNA method development.

Also, we note that although we already showed on the example of MI-GRAAL and IsoRankN that combining NCF of one method and AS of another method can lead to a new superior method [2, 3], testing whether the same holds for MI-GRAAL and GHOST, and in particular identifying the superior of the two NCFs, is of importance. First, validating that this also holds for MI-GRAAL and GHOST would only further stress out the need to carefully design a strategy for evaluating a novel approach against existing ones. Simply comparing the approaches, as has typically been done, is not enough. A more advanced evaluation strategy, such as our mix-and-match approach, is more appropriate. Second, MI-GRAAL’s NCF is a graphlet-based measure of topological node similarity [37] that is also used by many other network aligners [2, 3, 5, 6, 38] or even network clustering methods [37, 39, 40] to link network topology with biological function. When a new measure of topological similarity appears that is also argued to successfully capture biological function, such as GHOST’s NCF, it is extremely important to fairly compare it against the graphlet-based node similarity measure (which has not been done to date). In this way, future studies oriented towards learning new biological knowledge from network topology can focus on the most accurate node similarity measure. And this is exactly one of the goals of our study—to determine which of the two NCFs is superior. (We already demonstrated the superiority of MI-GRAAL’s graphlet-based NCF over IsoRankN’s popular PageRank-based NCF [2, 3].)

### Our approach and contributions

MI-GRAAL [7] and GHOST [25] are two state-of-the-art global network aligners that injectively map nodes between two networks in a way that preserves topologically or functionally conserved network regions. The two methods are conceptually similar, in the sense that their NCFs assume two nodes from different networks to be similar if their topological neighborhoods are similar. However, the mathematical and implementation details of the two NCFs are different. The same holds for the two methods’ ASs. To evaluate the contribution to the alignment quality of each of the two NCFs and two ASs, we mix and match these, resulting in a total of four different combinations. We then use each combination to produce alignments for synthetic networks with known ground truth node mapping as well as for real-world networks without known ground truth node mapping, and we evaluate the quality of each alignment with respect to five topological and two biological alignment quality measures.

In general, we find that MI-GRAAL’s NCF is superior to GHOST’s NCF, while the superiority of the methods’ ASs is data-dependent. Hence, for those network data on which GHOST’s AS is superior to MI-GRAAL’s AS, we propose the combination of MI-GRAAL’s NCF and GHOST’s AS as a new superior network aligner.

While fairly evaluating MI-GRAAL’s and GHOST’s NCFs and ASs, we approach two additional important research questions that, to our knowledge, have not been asked systematically in the context of network alignment thus far: (1) how much of the node similarity information within the NCF should come from protein sequence data compared to network topology data, and (2) how large the size of the neighborhoods of the compared nodes from different networks should be when generating topological similarity information within the NCF. Current GNA methods generally use a seemingly arbitrary amount of sequence information in their NCF, and also, they assume that the larger the size of a node’s neighborhood, the better the alignment quality. Thus, in this study, we evaluate whether these “state-of-the-art” choices are actually appropriate. We note that the first question has been recognized in some of the existing work [25, 31, 41], but this question has not been systematically addressed to the same extent as in our study. To our knowledge, the second question has not been addressed at all thus far.

In general, we find that which amount of sequence information is used within NCF does not drastically affect neither topological or biological alignment quality, while the effect of topological information is drastic. Namely, using no topological information within NCF results in poor topological and sometimes even biological alignment quality. Hence, topology takes precedence over sequence when it comes to improving alignment quality. Also, we find that using larger network neighborhood sizes within NCF in most cases leads to better alignment quality than using smaller neighborhood sizes. However, it is not always the case that the largest neighborhood size is the best; in many cases, the second largest size is the best. Therefore, using this size instead of the largest one would drastically decrease computational complexity of the given method without decreasing its accuracy.

We note that a recent study [31] performed a valuable survey of a number of GNA methods, focusing in the process on ranking the different methods based on their performance. However, that study did not focus on in-depth understanding why a given aligner performs the way it does, which is what we aim to do in our study. By analyzing a GNA method’s NCF and AS individually, we are able to understand the effect on alignment quality of each of the two algorithmic components. Furthermore, this existing study [31] compared the different methods with respect to a topological alignment quality measure called induced conserved structure (ICS) [25]. However, recently it was shown that ICS is an inappropriate measure of topological alignment quality, and a new superior measure was proposed, called symmetric substructure score ($S^3$) [30]. Here, we use the $S^3$ measure, along with several additional measures, thus increasing the confidence in our results compared to the results reported in Clark and Kalita [31]. In addition, this existing study [31] evaluated the different network aligners only on real-world networks of different species, for which the ground truth node mapping is *not known*. Here, we do the same, and we *also* align a high-confidence biological network to its noisy counterparts (“Data sets”). In the latter case, the ground truth node mapping is *known* and we can thus measure how well each aligner reconstructs the node mapping [corresponding to *node correctness* (“Network alignment quality measures”)]. This important evaluation cannot be done when the actual node mapping is not known and was thus not carried out in Clark and Kalita [31], despite the fact that measuring node correctness is the most appropriate way of *evaluating* a network aligner’s accuracy [5–7, 25, 30] before *applying* the aligner to networks of different species to learn new biological knowledge. Moreover, this existing study [31] still arrived to the conclusion that GHOST is “an excellent performer”, despite the fact that many newer methods were involved into the comparison. Thus, our results showing that we can improve GHOST even further by using its AS on top of MI-GRAAL’s NCF are an additional novel contribution of our study. Finally, we note again that in addition to providing comprehensive in-depth evaluation of the two prominent network aligners (rather than simply comparing their performance as in Clark and Kalita [31]), we also study in detail the effect of different parameters (such as the amount of sequence information or neighborhood size considered within NCF) on the alignment quality; this was not done in the recent study [31].

## Methods

### Data sets

We use two popular benchmark sets of networks in this study: (1) synthetic networks with known ground truth node mapping and (2) real-world protein–protein interaction (PPI) networks without known ground truth node mapping [2, 3, 7, 25, 30].

The synthetic network data with known node mapping consists of a high-confidence yeast PPI network, which has 1,004 proteins and 8,323 PPIs [5–7, 25, 30, 42], and five additional networks that add noise to the yeast network. Noise is the addition to the yeast network of low-confidence edges from the same data set [42], and each of the five additional noisy networks adds $x\%$ noise to the original network, where $x$ varies from 5 to 25% in increments of 5%. In this network set, we align the original yeast network to each of the synthetic networks with $x\%$ noise, resulting in the total of five network pairs to be aligned.

The real-world PPI network data without known node mapping consists of PPI networks of the following four species: *S. cerevisiae* (yeast/Y), *D. melanogaster* (fly/F), *C. elegans* (worm/W), and *H. sapiens* (human/H). The yeast, fly, worm, and human networks have 3,321 proteins and 8,021 PPIs, 7,111 proteins and 23,376 PPIs, 2,582 proteins and 4,322 PPIs, and 6,167 proteins and 15,940 PPIs, respectively [43]. In this network set, we align PPI networks for each pair of species, resulting in the total of six network pairs to be aligned.

We note that the synthetic network data is not truly synthetic, as both the original yeast network and the noise in terms of the lower-confidence PPIs come from an actual experimental study [42]. We refer to this network set as synthetic simply because we know the known ground truth node mapping, unlike for the real-world PPI network set. Also, we note that the synthetic network data encompasses “co-complex” PPIs obtained by affinity purification followed by mass-spectrometry (AP/MS), among other PPI types, while the real-world PPI network data consists of “binary” yeast two-hybrid (Y2H) PPIs. Another difference between the two network sets is that for the synthetic data the smaller (original yeast) network is an exact subgraph of the larger (noisy) network, whereas this is not the case for networks of different species in the real-world data.

When evaluating the amount of sequence data that should be used within NCF when generating an alignment, we use protein sequence similarity data. This data set comes from BLAST bit-values from the NCBI database [44].

When evaluating the biological alignment quality with respect to functional enrichment of the aligned nodes, we use Gene Ontology (GO) annotation data from our recent study [2, 3].

Importantly, we note that we use the above data sources and versions of the data because the exact same data have already been used in the existing work, which allows for fair and consistent method evaluation. If the main focus of one’s work was to predict new biological knowledge rather than to conduct fair method evaluation and comparison, then we would recommend using the latest and thus most complete versions of the data.

### Existing network aligners and their NCFs and ASs

#### MI-GRAAL’s NCF

MI-GRAAL improves upon its predecessors, GRAAL [5] and H-GRAAL [6], by using the same NCF (see below) but by combining GRAAL’s and H-GRAAL’s ASs into a new superior AS (see below).

**Figure 1:**
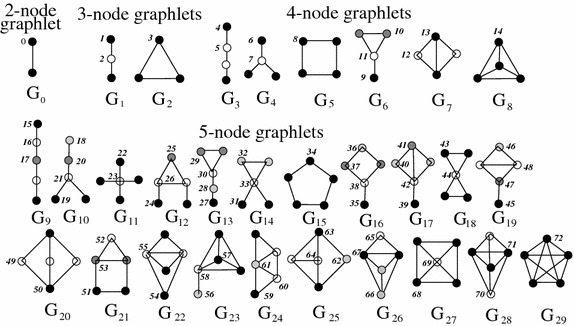
Illustration of MI-GRAAL’s NCF. To compute topological node similarities, this NCF relies on thirty 2-, 3-, 4-, and 5-node graphlets $G_0, G_1,\ldots, G_{29}$ and their “node symmetry groups”, also called automorphism orbits, numbered 0, 1, 2,…, 72. In a graphlet $G_i$, $i \in \{0,1,\ldots,29\}$, nodes belonging to the same orbit are of the same shade. For details, see the original publication [49].

MI-GRAAL’s NCF relies on the concept of small induced subgraphs called * graphlets* (Figure 1) [37, 39, 40, 45–47]. All 2–5-node graphlets are considered. Because of the small-world nature of real-world networks, using larger graphlets would unnecessarily increase the computational complexity needed the count the graphlets [5, 6]. Based on the graphlets, the *node graphlet degree vector* (node-GDV) is computed for each node in each network, which counts how many times the given node touches each of the 2–5-node graphlets, i.e., each of their 73 node symmetry groups (or *automorphism orbits*; Figure 1). As such, node-GDV captures up to a four-deep network neighborhood of the node of interest. By comparing node-GDVs of two nodes to compute their *node-GDV-similarity*, and by doing so between each pair of nodes in different networks, one is able to capture pairwise topological node similarities between the different networks.

MI-GRAAL also allows for integration of other node similarity measures into its NCF, such as protein sequence similarity. Thus, MI-GRAAL has the built-in functionality of allowing the user to incorporate their own custom pairwise node scores rather than rely on MI-GRAAL’s NCF, which is exactly how we incorporate GHOST’s NCF as input into MI-GRAAL’s AS.

#### MI-GRAAL’s AS

GRAAL’s AS utilizes a seed-and-extend approach to greedily maximize the total NCF over all aligned nodes. H-GRAAL, on the other hand, finds optimal alignments with respect to the total NCF by using the Hungarian algorithm to solve the linear assignment problem. MI-GRAAL’s AS combines GRAAL’s greedy seed-and-extend approach with H-GRAAL’s optimal AS into a superior AS.

Specifically, for graphs $G$ and $H$, MI-GRAAL’s AS selects a pair of nodes $u$ and $v$, where $u \in G$ and $v \in H$, which have the highest similarity score among all pairs of nodes from the different networks. It then begins to align these nodes’ neighbors as follows. Let us denote by $N_G(u)$ and $N_H(v)$ the sets of neighbors of nodes $u$ and $v$, respectively. A bipartite graph is constructed using nodes from $N_G(u)$ and $N_H(v)$, where there exists an edge between a node $x$ from $N_G(u)$ and a node $y$ from $N_H(v)$ if and only if a neighbor of $x$ has already been aligned to a neighbor of $y$. A confidence weight (i.e., the NCF-based similarity between two given nodes) is then assigned to each edge. Given the resulting bipartite graph, MI-GRAAL’s AS solves the maximum weight bipartite matching problem to determine which nodes in $N_G(u)$ and $N_H(v)$ should be aligned to each other. After MI-GRAAL’s AS is done aligning nodes from $N_G(u)$ to nodes from $N_H(v)$, it then expands to these nodes’ neighbors and repeats the above steps to align them. The expansion continues iteratively until the entire smaller network is exhausted. For more details on MI-GRAAL’s AS, see the original publication [7].

#### GHOST’s NCF

**Figure 2:**
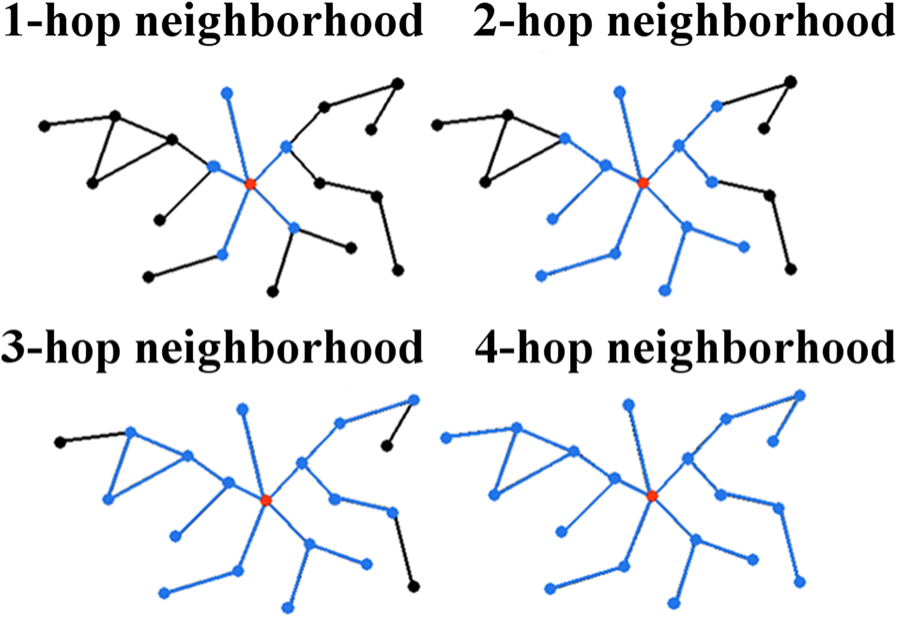
Illustration of GHOST’s NCF. To compute topological node similarities, this NCF compares two nodes in different networks with respect to similarity of each of their $k$-hop neighborhoods, $k=1,2,3,4$. All *blue* edges and * blue* nodes are within the given $k$-hop neighborhood of the *red* node.

GHOST’s NCF takes into account a node’s $k$-hop neighborhood $(k=4)$, which is the induced subgraph on all nodes whose shortest path distance from the node in question is less than or equal to $k$ (Figure  2). Intuitively, GHOST’s NCF computes topological distance (or equivalently similarity) between two nodes from different networks by comparing the nodes’ “spectral signatures”. These signatures are based on the spectrum of the normalized Laplacian for subgraphs of radius $k$ centered around a given node. Essentially, the spectral signature of a node is based on subgraph counts in the node’s k-hop neighborhood [25]. GHOST also allows for the incorporation of sequence information into its NCF, in which the resulting NCF is a linear combination of GHOST’s topological and sequence distance scores. For further details on GHOST’s NCF, refer to the original publication [25]. In our study, we consider $k=1, 2, 3, 4$, which allows for a fair comparison of GHOST’s NCF to MI-GRAAL’s NCF when varying the size of network neighborhood that is considered within the NCFs (“Aligners resulting from combining existing NCFs and ASs, and their parameters”).

#### GHOST’s AS

**Figure 3:**
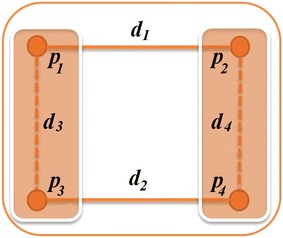
Intuitive comparison of MI-GRAAL’s and GHOST’s ASs. Let us assume that we are aligning two graphs $G_1(V_1,E_1)$ and $G_2(V_2,E_2).$ Let $p_1,p_3 \in V_1$, let $p_2,p_4 \in V_2$, and let the NCF distance (equivalently, similarity) between the node pairs be $d_1,d_2,d_3,d_4$, as illustrated. MI-GRAAL’s alignment strategy only considers the values $d_1$ and $d_2$ when creating an alignment, while GHOST’s AS considers the values $d_1,d_2,d_3,{\text{and }}d_4$ when doing so.

GHOST’s AS is also a seed-and-extend method, but unlike MI-GRAAL’s AS that deals with the linear assignment problem, GHOST’s AS deals with the quadratic assignment problem (Figure 3 illustrates this). GHOST’s AS uses a two-phase seed-and-extend strategy by first selecting nodes $u$ and $v$, where $u \in G$ and $v \in H$, which have the highest similarity score among all pairs of nodes from the different networks, and then extending around these nodes to align their neighbors [i.e., nodes from $N_G(u)$ and $N_H(v)$]. To do this, GHOST’s AS considers pairwise similarities between nodes in $N_G(u)$ and $N_H(v)$ in addition to similarities between nodes within the same network, and all of these similarities are used to estimate a solution to the quadratic assignment problem, which is the node alignment. For further details on GHOST’s AS, refer to the original publication [25].

### Aligners resulting from combining existing NCFs and ASs, and their parameters

#### Mixing and matching different NCFs and ASs

To fairly evaluate the two algorithmic components of MI-GRAAL and GHOST, we aim to first compare the two NCFs under the same AS, for each of the two ASs. We then aim to compare the two ASs under the same NCF, for each of the two NCFs. This results in a total of four aligners, i.e., different combinations of the two methods’ NCFs and ASs. However, GHOST does not allow the user to import their own (e.g., MI-GRAAL’s) NCF into its AS, so we are unable to study the combination of MI-GRAAL’s NCF and GHOST’s AS. Thus, in total, we consider three different aligners (Table  1).

**Table 1:** The three aligners considered in this study

Aligner	Node cost function	Alignment strategy
M-M	MI-GRAAL	MI-GRAAL
G-M	GHOST	MI-GRAAL
G-G	GHOST	GHOST

The first letter in the aligner represents NCF of the aligner, while the second letter represents AS of the aligner.

#### Varying the amount of sequence versus topological information within NCF

An additional goal of this paper is to determine the most appropriate amount of sequence information versus topological information to be included into NCF. Thus, for each aligner, we generate NCFs with varying amounts of sequence and topology information, as $\alpha T + (1-\alpha )S,$ where $T$ represents topological similarity score (e.g. node-GDV-similarity) and $S$ represents sequence similarity score. We vary $\alpha$ from 0 to 1 in increments of 0.1.

#### Varying the size of network neighborhood within NCF

Further, we aim to determine the most appropriate neighborhood size that should be used within NCF when producing an alignment. Thus, for each aligner (and for each value of $\alpha$), we also consider four different neighborhood sizes, as described in Table 2. We note that although we have tried to classify under the same neighborhood size label (e.g. T1 in Table 2) graphlet sizes considered within MI-GRAAL’s NCF and $k$-hop values considered within GHOST’s NCF, it is not necessarily the case that the neighborhood of a node that is covered by graphlets of a given size and the neighborhood of the same node that is covered by the corresponding $k$-hop value match exactly. That is, for example, 2–3-node graphlets and 2-hop neighborhood (both corresponding to T2 in Table 2) do not necessarily cover exactly the same amount of network topology. Yet, we have aimed to provide as accurate as possible classification in Table 2, in order to allow for as fair as possible comparison of the two methods’ NCFs under varying sizes of network neighborhoods.

**Table 2:** The four neighborhood sizes that we vary within each aligner

Neighborhood size	Graphlet size (used by MI-GRAAL’s NCF)	${\bf k}$-hop neighborhood (used by GHOST’s NCF)
T1	2-node graphlets	1-hop neighborhood
T2	2–3-node graphlets	2-hop neighborhood
T3	2–4-node graphlets	3-hop neighborhood
T4	2–5-node graphlets	4-hop neighborhood

#### Implementation details

The types of scores that MI-GRAAL and GHOST take in as input are different: MI-GRAAL looks at node similarities (the higher the score, the more similar the nodes), while GHOST looks at node distances (the lower the score, the more similar the nodes). We carefully take this into account to allow for fair method comparison. For example, to ensure that neither NCF has an advantage due to the format of the scores, we normalize all scores. That is, node similarity scores used in MI-GRAAL can exceed the value one, while no scores generated by GHOST are greater than one. To make the two sets of scores comparable, we scale MI-GRAAL’s node similarity scores to the [0–1] range by dividing each of the scores by the maximum similarity score. Because GHOST deals with distances rather than similarities, we take one minus GHOST's NCF and then plug in the resulting node scores into MI-GRAAL's AS.

Further, MI-GRAAL’s NCF returns all pairwise node similarity scores between two networks. However, GHOST’s NCF returns only a subset of all pairwise node distance scores, depending on the network size. To complete GHOST’s pairwise node score matrix and thus allow for it to be given as input into MI-GRAAL’s AS, we assign a score equal to the highest distance score returned by GHOST to all node pairs for which GHOST did not return a distance score.

Finally, the current implementation of MI-GRAAL’s AS does not function properly when a large pairwise node similarity matrix is plugged into it. Thus, MI-GRAAL’s AS has had difficulty aligning the two largest networks from our study, the fly and human networks. As a solution, we create a matrix that contains only the top 21 million node similarity scores of the original node similarity matrix, this being the maximum that our computational resources would process. With this adjustment, we are successfully able to generate all fly-human alignments.

### Network alignment quality measures

We use well established network alignment quality measures [2, 3, 30]. Let $G_1(V_1,E_1)$ and $G_2(V_2,E_2)$ be two graphs such that $|V_1| \le |V_2|$. An alignment of $G_1$ to $G_2$ is a total injective function $f : V_1 \rightarrow V_2$; every element of $V_1$ is matched uniquely with an element of $V_2$. Let us denote by $E^{\prime}_2$ the set of edges from $G_2$ that exist between nodes in $G_2$ that are aligned by $f$ to nodes in $G_1$.

#### Topological evaluation

We use five measures of topological alignment quality:

1. *Node correctness* (*NC*) If $h : V_1 \rightarrow V_2$ is the correct ground truth node mapping between $G_1$ and $G_2$ (when such mapping is known), then NC of alignment $f$ is: $NC = \frac{|\{u \in V_1 : h(u) = f(u) \}|}{|V_1|} \times {100\%}$ [5]. This measure can be computed only for alignments of the synthetic network set with known ground truth node mapping (“Data sets”). All remaining measures (listed below) can be computed for the real network set with unknown node mapping as well.
2. *Edge correctness* (*EC*) EC is the percentage of edges from $G_1$, the smaller network (in terms of the number of nodes), which are aligned to edges from $G_2$, the larger network [5]. Formally, $EC = \frac{|E_{1} \cap E'_2|}{|E_{1}|}\times {100\%},$ where the numerator is the number of “conserved” edges, i.e., edges that are aligned under the given node mapping. The larger the EC score, the better the alignment.
3. *Induced conserved structure* (*ICS*) $ICS = \frac{|E_{1} \cap E'_2|}{|E'_2|}\times {100\%}.$ EC might fail to differentiate between alignments that one might intuitively consider to be of different topological quality [25], since it is defined with respect to edges in $E_1$. For example, aligning a $k$-node cycle in $G_1$ to a $k$-node cycle in $G_2$ would result in the same EC as aligning a $k$-node cycle in $G_1$ to a $k$-node clique (complete graph) in $G_2.$ Clearly, the former is intuitively a better alignment than the latter, since no edges that exist between the $k$ nodes in $G_2$ are left unaligned in the first case, whereas many edges are left unaligned in the second case. Since ICS is defined with respect to edges in $E'_2$, it would have the maximum value of 100% when aligning a $k$-node cycle to a $k$-node cycle, and it would have a lower value when aligning a $k$-node cycle to a $k$-node clique [30]. The larger the ICS, the better.
4. *Symmetric substructure score* ($S^3$) EC penalizes the alignment for having misaligned edges in the smaller network. ICS penalizes the alignment for having misaligned edges in the larger network. S$^3$ on the other hand, aims to improve upon EC and ICS by penalizing for misaligned edges in both the smaller and larger network. S$^3 = \frac{|E_{1} \cap E'_2|}{|E_1| +|E'_2| - |E_{1} \cap E'_2|}\times {100\%}.$ For details, see the original publication [30].
5. The size of the *largest connected common subgraph* (*LCCS*) [5], which we use for the following reason. Of two alignments with similar EC, ICS, or S$^3$ scores, one could expose large, contiguous, and topologically complex regions of network similarity, while the other could fail to do so. Thus, in addition to counting aligned edges or nodes that participate in the aligned edges, it is important that the aligned edges cluster together to form large connected subgraphs rather than being isolated. Hence, we define a connected common subgraph (CCS) as a connected subgraph (not necessarily induced) that appears in both networks [6]. We measure the size of the largest CCS (LCCS) in terms of the number of nodes as well as edges. Namely, we compute the LCCS score as in our recent work [30]. First, we count $N$, the percentage of nodes from $G_1$ that are in the LCCS. Then, we count $E$, the percentage of edges that are in the LCCS out of all edges that could have been aligned between the nodes in the LCCS. That is, $E$ is the minimum of the number of edges in the subgraph of $G_1$ that is induced on the nodes from the LCCS, and the number of edges in the subgraph of $G_2$ that is induced on the nodes from the LCCS [30]. Finally, we compute their geometric mean as $\sqrt{(}N \times E)$, in order to penalize alignments that have small $N$ or small $E$. Large values of this final LCCS score are desirable.

#### Biological evaluation

Only alignments in which many aligned node pairs perform the same function should be used to transfer function from annotated parts of one network to unannotated parts of another network [30]. Hence, we measure GO [48] enrichment of aligned proteins pairs, i.e., the percentage of protein pairs in which the two proteins *share* at least one GO term, out of all aligned protein pairs in which both proteins are annotated with at least one GO term. We refer to this percentage as *GO correctness* (*GO*). We do this with respect to complete GO annotation data, independent of GO evidence code. Also, since many GO annotations have been obtained via sequence comparison, and since some of the aligners use sequence information, we repeat the analysis considering only GO annotations with experimental evidence codes, in order to avoid the circular argument. In this case, we refer to GO correctness as *experimental GO correctness* (*EXP*). The higher the GO and EXP values, the better [30].

## Results and discussion

We aim to answer the following three main questions in the context of network alignment: (1) which NCF and AS is superior to the other, and is there perhaps a combination of one existing method’s NCF and another existing method’s AS that is the superior aligner in terms of accuracy as well as time complexity (“What is the best NCF and the best AS?”)? (2) How much sequence versus topological information to use within NCF (“The amount of sequence versus topological information within NCF?”)? (3) How large the size of network neighborhoods of compared nodes to consider within NCF (“The size of nodes’ neighborhoods within NCF?”)? In addition, we comment on relationships between different alignment quality measures (“Relationships between different alignment quality measures”). Finally, we conclude in “Conclusions”.

### What is the best NCF and the best AS?

By comparing M-M and G-M aligners, we can fairly compare the two NCFs under the same (MI-GRAAL’s) AS. Also, by comparing G-M and G-G, we can fairly compare the two ASs under the same (GHOST’s) NCF. See “Aligners resulting from combining existing NCFs and ASs, and their parameters” for details on each aligner.

#### Synthetic networks with known node mapping

**Figure 4:**
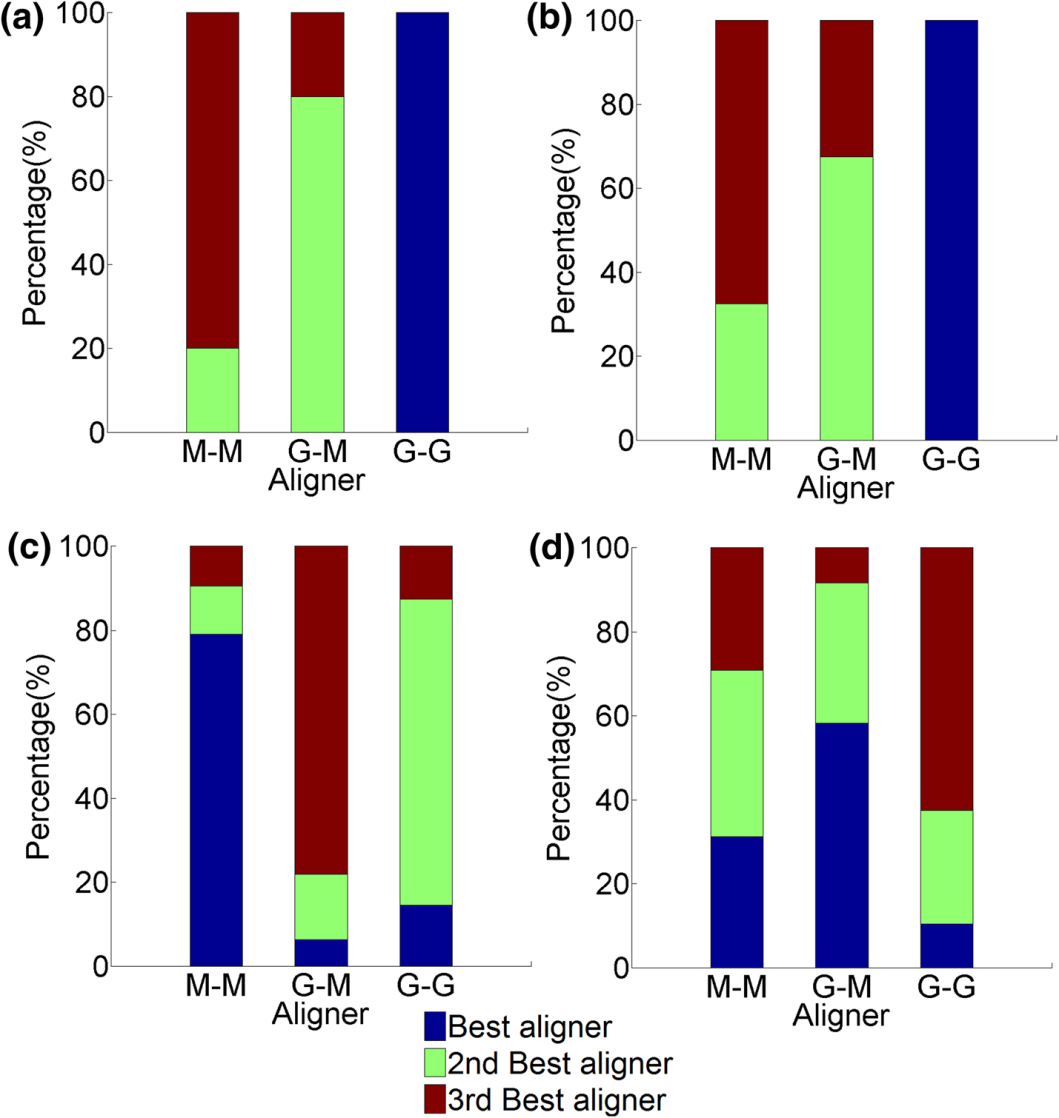
The ranking of the three aligners (M-M, G-M, and G-G). The ranking is shown over *all* alignments for all values of $\alpha$ and all neighborhood sizes, with respect to: **a** all topological scores of all alignments with known ground truth node mapping, **b** all biological scores of alignments with known node mapping, **c** all topological scores of alignments with unknown node mapping, and **d** all biological scores of alignments with unknown node mapping.* Percentages* represent the percentage of cases that an aligner achieved a certain ranking.

**Figure 5:**
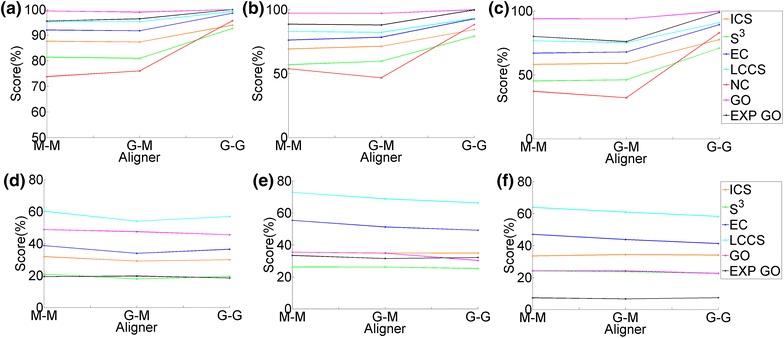
Alignment quality results of the three aligners (M-M, G-M, and G-G). The results are shown for *best* alignments over all values of $\alpha$ and all neighborhood sizes, for **a**–**c** three network pairs with known node mapping (yeast–yeast 5%, yeast–yeast 10%, and yeast–yeast 15%, respectively) and **d**–**f** three network pairs with unknown mapping (human–yeast, human–worm, and worm–yeast, respectively).* Percentages* represent the scores achieved by an alignment quality measure. For equivalent results for the remaining network pairs, see the Additional file 1: Figures S1–S2 and S8–S10.

Overall, GHOST’s NCF is slightly superior to that of MI-GRAAL (Figure 4a, b). Also, GHOST’s AS is superior to MI-GRAAL’s AS (Figure 4a, b). However, these findings are based on *all* alignments (with known node mapping) for all values of $\alpha$, all neighborhood sizes, and all measures of alignment quality combined (“Aligners resulting from combining existing NCFs and ASs, and their parameters”), which might not be fair. Thus, in Figure 5a–c, for each aligner, for each alignment quality measure, we show results for the *best* alignments over all values of $\alpha$ and all neighborhood sizes, for three out of all five network pairs (for the remaining network pairs, see Additional file 1: Figures S1 and S2). Now, the general trend (and especially with respect to NC as the most accurate ground truth measure of alignment quality) is that the best scores for M-M are either comparable or superior to those of G-M, indicating slight superiority of MI-GRAAL’s NCF over GHOST’s. Nonetheless, G-G still always outperforms G-M, indicating superiority of GHOST’s AS over MI-GRAAL’s AS.

It is possible to break down the above results and study how the ranking of the different NCFs and ASs changes with the change in the value of $\alpha$, which corresponds to the amount of topological similarity information used within NCF (Additional file 1: Figures S3–S7). In general, MI-GRAAL’s NCF is comparable to GHOST’s NCF across all $\alpha$ values, as M-M and G-M scores are similar. On the other hand, GHOST’s AS shows superiority over MI-GRAAL’s AS, as G-G consistently results in higher scores than G-M. We note that we show that the value of $\alpha$ does not greatly affect alignment quality (“The amount of sequence versus topological information within NCF?”).

It is also possible to break down the above results even further and study how the ranking of the different NCFs and ASs changes with the change in the neighborhood size that is considered within NCF (Additional file 1: Figures S3–S7). In general, for the smaller neighborhood sizes (T1 and T2), GHOST’s NCF generally produces comparable or superior results to MI-GRAAL’s NCF, as G-M scores are higher than M-M scores. However, for the larger neighborhood sizes (T3 and T4), MI-GRAAL’s NCF is comparable or superior to GHOST’s NCF. And because we show that the larger neighborhood sizes (T3 and T4) are overall superior (“The size of nodes’ neighborhoods within NCF?”), this means that overall MI-GRAAL’s NCF is comparable to or superior to GHOST’s NCF. On the other hand, in general, for all network sizes, GHOST’s AS consistently outperforms MI-GRAAL’s AS, as G-G scores is typically higher than G-M scores.

**Figure 6:**
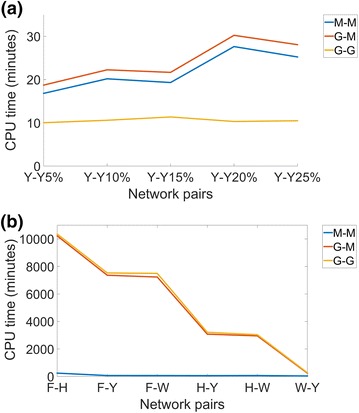
The CPU time needed for M-M, G-M, and G-G (when using neighborhood size T4) to generate alignments of: **a** the synthetic noisy yeast networks and **b** the real-world networks of different species. All experiments were run on the same server with 16 2.3 GHz processors and 128 GB of RAM.

When comparing the different aligners with respect to computational complexity (rather than accuracy, as above), we find the following. Overall, G-G is the fastest, followed by M-M, followed by G-M (Figure 6a). This implies that since M-M is faster than G-M, MI-GRAAL’s NCF is less computationally intensive than GHOST’s NCF. Also, since G-G is faster than G-M, GHOST’s AS is less computationally intensive than MI-GRAAL’s AS.

We note that in order to fairly compare the running times of all aligners used in this study, we run all aligners using neighborhood size T4 (Table 2). We cannot do this for the other (smaller) neighborhood sizes for the following reasons. While GHOST allows the user to specify any desired neighborhood size as input, MI-GRAAL’s NCF does not. Namely, the current implementation of MI-GRAAL by default computes all up to 5-node graphlets (i.e., T4). Then, to get the information contained in up to 2-, 3-, or 4-node only graphlets, one simply considers the relevant dimensions of the entire up to 5-node graphlet degree vector and discards all other dimensions. Thus, we cannot evaluate the computational complexity of considering 2-, 3-, or 4-node only graphlets, as with the current implementation, each of these options takes the same (longest) amount of time that computing up to 5-node graphlets takes.

#### Real networks with unknown node mapping

Overall, unlike for the synthetic network data set with known node mapping, on the real network data set with unknown mapping, MI-GRAAL’s NCF is now comparable or superior to that of GHOST (Figure 4c, d). Further, MI-GRAAL’s AS is now comparable or superior to GHOST’s AS (Figure 4c, d). We confirm these findings even when we limit from *all* alignments (Figure 4c, d) to the *best* alignments only (just as above) (Figure 5d–f) (Additional file 1: Figures S8–S10).

When zooming into the results further to observe the effect of the $\alpha$ parameter, in general, for all values of $\alpha$, MI-GRAAL’s NCF is comparable or superior to GHOST’s NCF and MI-GRAAL’s AS is comparable to GHOST AS across all values of $\alpha$ (Additional file 1: Figures S11–S16). The same holds independent on the neighborhood size that is considered within NCF (Additional file 1: Figures S11–S16).

When comparing the different aligners with respect to computational complexity (rather than accuracy, as above), we find the following. Unlike for the synthetic network data, we now observe that M-M is significantly the fastest, followed by G-M, followed by G-G (Figure 6b). This implies that since M-M is faster than G-M, MI-GRAAL’s NCF is less computationally intensive than GHOST’s NCF. Also, since G-M is faster than G-G, MI-GRAAL’s AS is less computationally intensive than GHOST’s AS.

**Figure 7:**
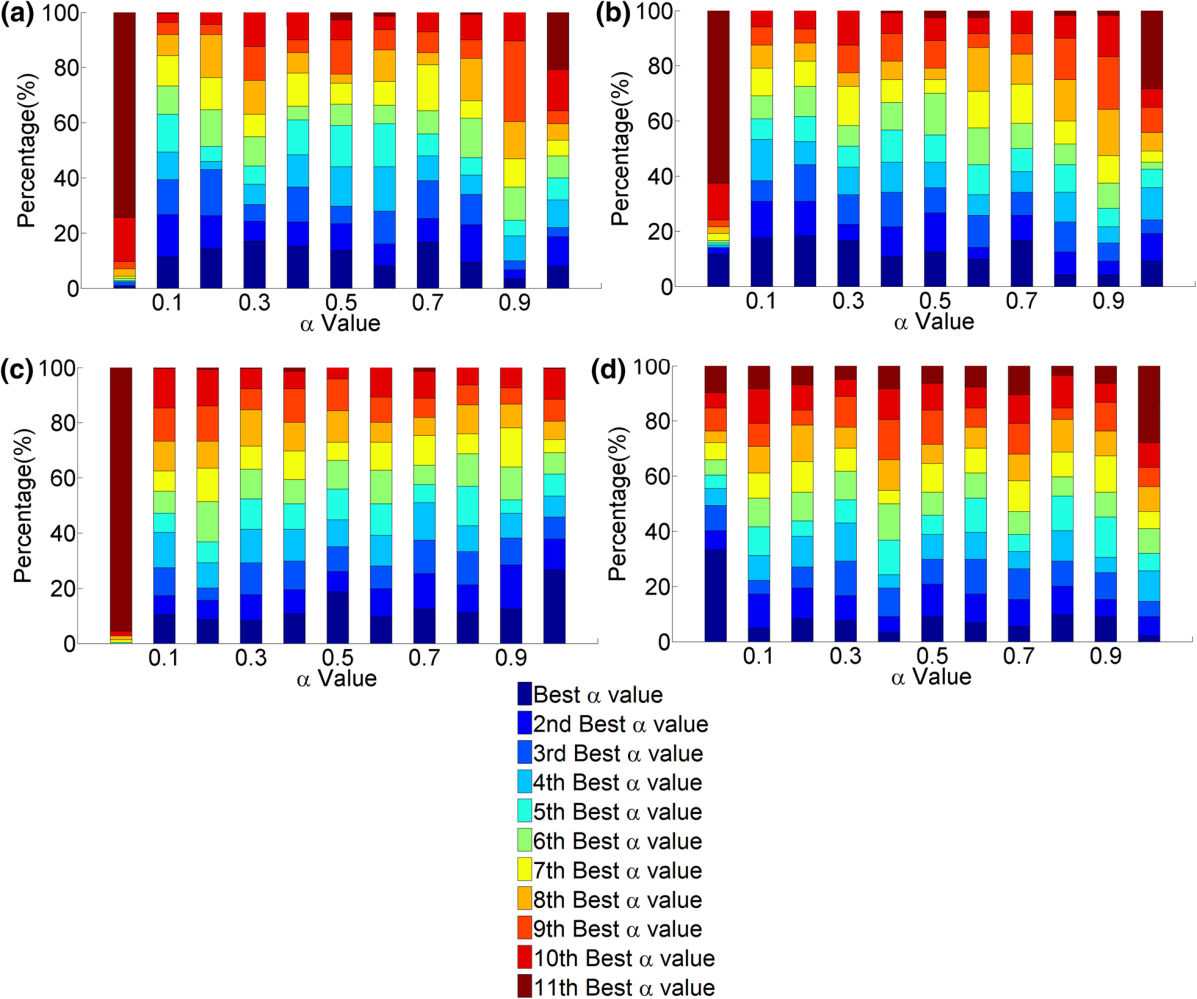
The ranking of the 11 values of $\alpha$ (from 0 to 1 in increments in 0.1). The ranking is shown over *all* alignments for all aligners and all neighborhood sizes, with respect to: **a** all topological scores of alignments with known ground truth node mapping, **b** all biological scores of alignments with known node mapping, **c** all topological scores of alignments with unknown node mapping, and **d** all biological scores of alignments with unknown node mapping.* Percentages* represent the scores achieved by an alignment quality measure.

#### Summary

Which NCF or AS is the best *overall* is not easy to determine, as the results are data-dependent. But when we limit analyses of each aligner to the *best* alignments over all parameters, M-M is comparable or superior to G-M, indicating that MI-GRAAL’s NCF is better than GHOST’s NCF, while the performance of G-M versus G-G, i.e., of MI-GRAAL’s AS versus GHOST’s AS, is still data-dependent. These results hold not just in terms of accuracy but also in terms of computational complexity. We note that the reason why the performance of the two ASs is data-dependent (GHOST’s AS performing better on the synthetic networks, and MI-GRAAL’s AS performing better on the real-world networks) could be due to the differences of the two network data sets. Namely, recall that the synthetic network data encompasses “co-complex” PPIs obtained by AP/MS, among other PPI types, while the real-world network data consists of “binary” Y2H PPIs (“Data sets”).

The above results imply that the graphlet-based measure of topological node similarity [37] that MI-GRAAL uses (along with many other network aligners [2, 3, 5, 6] or even network clustering methods [37, 39, 40]) remains the state-of-the-art, as it is superior to the newer spectral signature-based node similarity measure that GHOST uses (and especially to the PageRank-based node similarity measure that aligners from the IsoRank family use, as we already showed in our recent study [2, 3]). Our results indicate that the slight superiority of GHOST (i.e., G-G) over MI-GRAAL (i.e., M-M) that was claimed in the original GHOST publication [25] seems to come from GHOST’s AS and *not* its NCF, which is not surprising, since GHOST’s AS deals with the quadratic assignment problem whereas MI-GRAAL’s AS deals only with linear assignment problem. Further, our results indicate that the combination of MI-GRAAL’s NCF and GHOST’s AS (i.e., M-G) could be a new aligner that is superior to the existing MI-GRAAL (i.e., M-M) and GHOST (ie., G-G) aligners on at least some data sets. Unfortunately, explicitly testing this is not possible with the current implementation of GHOST, as per our conversation with the authors of GHOST, the current implementation is too complex to modify to allow for plugging MI-GRAAL’s (or any other method’s) NCF into GHOST’s AS.

### The amount of sequence versus topological information within NCF?

Recall that we vary the amount of topological node similarity information within NCF with the $\alpha$ parameter (where $\alpha$ of 0 means that no topology information is used, i.e., that only sequence information is used, whereas $\alpha$ of 1 means that only topology information is used; “Aligners resulting from combining existing NCFs and ASs, and their parameters”). Here, we study the effect of the $\alpha$ parameter on alignment quality.

#### Synthetic networks with known node mapping

Overall, the value of $\alpha$ does not affect alignment quality, as long as some amount of topological information is used. That is, only $\alpha =0.0$ results in completely inferior alignments, especially with respect to topological alignment quality, whereas all other values of alpha are more-less comparable (Figure 7a, b).

It is expected that the larger the value of $\alpha$, i.e., the more of topological information is used within NCF, the better the topological alignment quality. Again, this is exactly what we observe (Figure 7a). It is also expected that the smaller the value of $\alpha$, i.e., the more of sequence information is used within NCF, the better the biological alignment quality. Surprisingly, this is not what we observe (Figure 7b): larger values of $\alpha$ (e.g., 0.7) result in more of high-quality alignments than $\alpha =0$.

**Figure 8:**
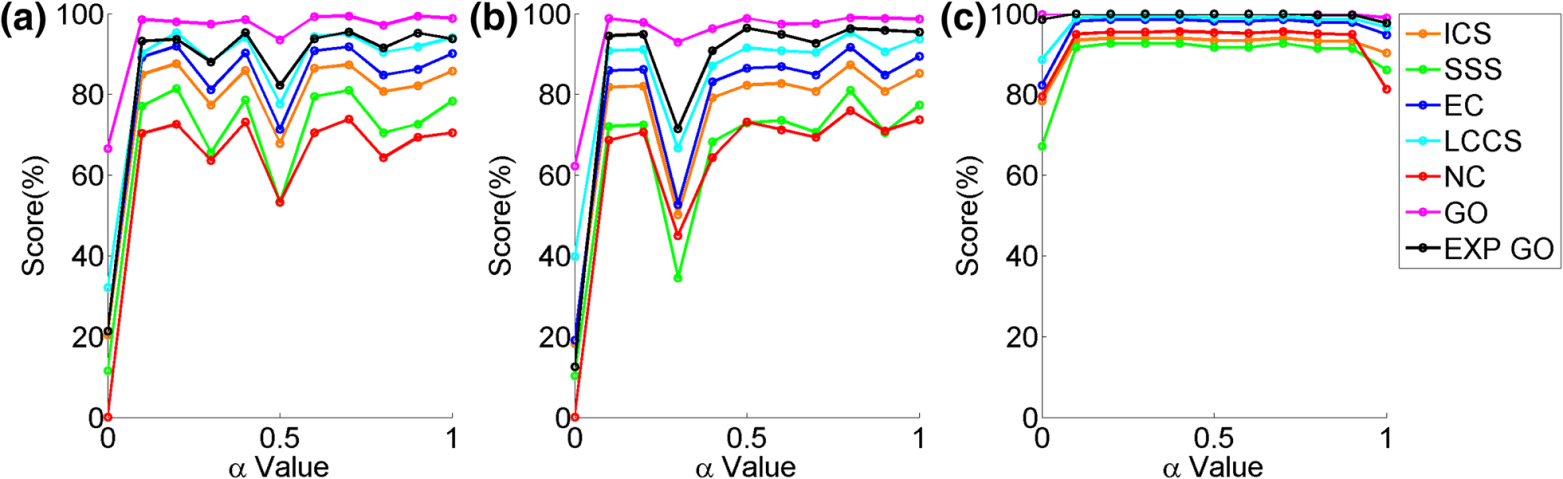
Detailed illustration of the effect of the $\alpha$ parameter for **a** M-M, **b** G-M, and **c** G-G aligners. In particular, results are shown for the yeast-yeast 5% alignment and for the neighborhood size T4.* Percentages* represent the scores achieved by an alignment quality measure. For other network pairs and other neighborhood sizes, see Additional file 1: Figures S3–S7 for synthetic network data and see see Additional file 1: Figures S11–S16 for real-world PPI network data.

When zooming into the results further to observe the effect of the aligner, in general, we see the same trends as above independent of the aligner (Additional file 1: Figures S3–S7). Namely, the results from Figure 7a, b hold independent on which NCF or AS is used. Further, there is no difference in the results across the two NCFs (Figure 8a, b). There is only a minor difference in the results across the two ASs, in the sense that the results are somewhat more stable across different $\alpha$s for GHOST’s AS than for MI-GRAAL’s AS (Figure 8b, c). Also, GHOST’s AS suggests that in addition to not using $\alpha =0$ (i.e., sequence alone), one should not use $\alpha =1$ either (i.e., topology alone); but other than that, the choice of $\alpha$ still has no major effect (Figure 8c).

When zooming into the results from Figure 7a, b further to observe the effect of the neighborhood size, we see that the results hold independent of the neighborhood size (Additional file 1: Figures S3–S7).

#### Real networks with unknown node mapping

The results that we observe for the synthetic networks in general hold for this network set as well. Namely, $\alpha =0$ results in the worst topological alignment quality, while the other $\alpha$ values are somewhat comparable, with a slight dominance of the larger values, as expected (Figure 7c). Interestingly, for this network set, the lowest value of $\alpha =0$ results in the most of highest-scoring alignments with respect to biological alignment quality; yet, even the largest $\alpha$s often lead to good alignments with respect to biological alignment quality (Figure 7d).

When zooming into the results further to observe the effect of the aligner, as with synthetic networks, the general results from Figure 7c, d hold independent of the aligner for real networks as well (Additional file 1: Figures S11–S16). However, unlike for synthetic networks, for real networks we now see result stability across all NCFs and all ASs, and not just for GHOST’s AS. Also, GHOST’s AS no longer suggests that $\alpha =1$ should not be used.

When zooming into the results from Figure 7c, d further to observe the effect of the neighborhood size, just as with the synthetic networks, we again see that the results hold independent of the neighborhood size (Additional file 1: Figures S11–S16).

#### Summary

Overall, at least some amount of topological information should be included within NCF, as this results in good topological as well as biological alignment quality. While $\alpha =0.0$ may (but does not always) result in biologically high-quality alignments, in every case it fails to produce topologically superior results. Thus, $\alpha =0.0$ should not be used.

### The size of nodes’ neighborhoods within NCF?

Intuitively, one would expect that the increase in the size of nodes’ network neighborhoods within NCF (i.e., in the amount of network topology) would result in higher-quality alignments. However, this assumption has not been tested to date. Instead, the existing methods blindly use the largest neighborhood size that is allowed by available computational resources (that is, MI-GRAAL uses all 2–5-node graphlets, whereas GHOST uses $k=4;$ “Aligners resulting from combining existing NCFs and ASs, and their parameters”). Thus, within each aligner, we vary the neighborhood size from T1 to T4 (Table 2) to systematically evaluate the effect of this parameter.

#### Synthetic networks with known node mapping

**Figure 9:**
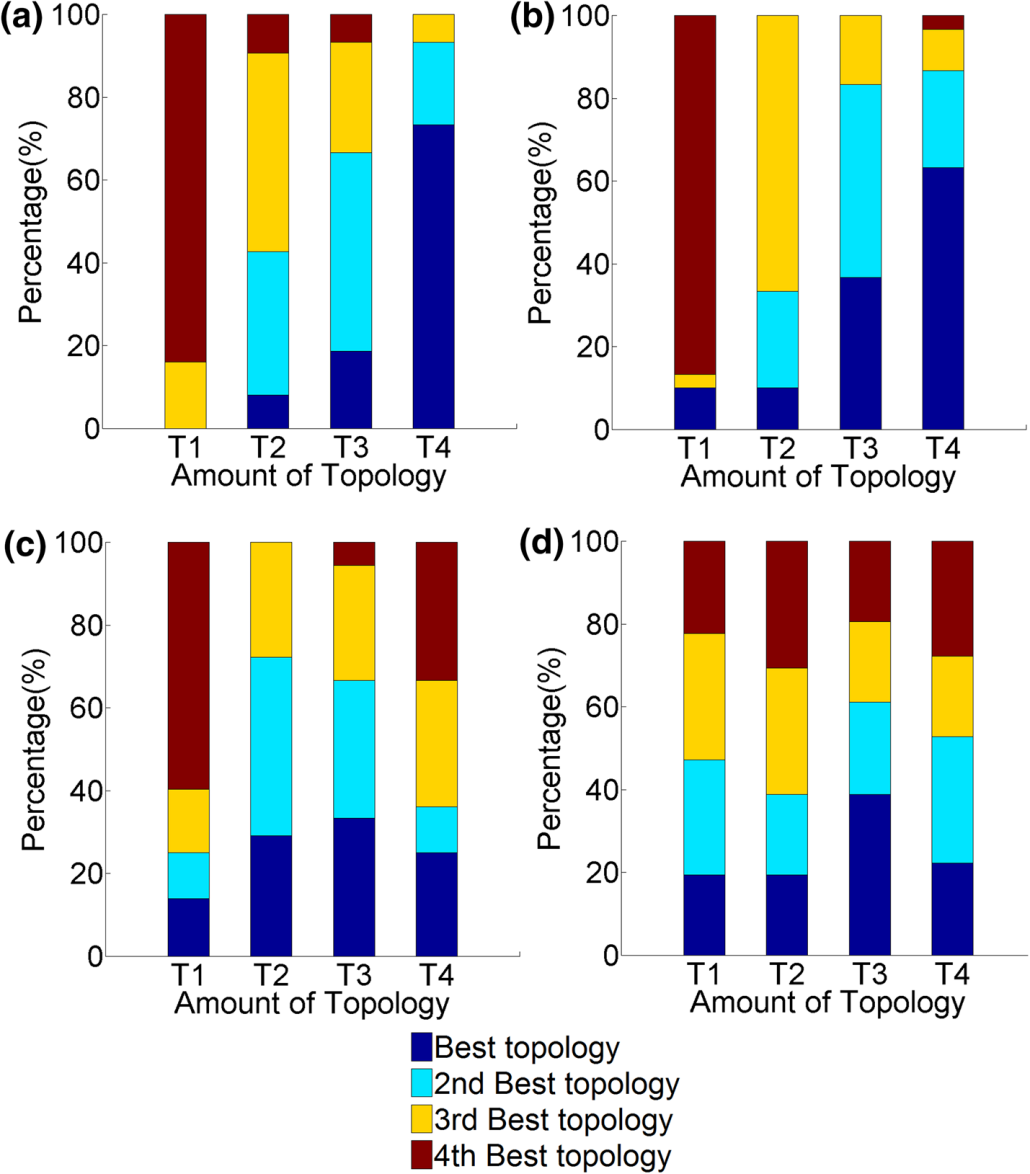
The ranking of the four neighborhood sizes (T1–T4). The ranking is shown over *all* alignments for all aligners and all values of $\alpha$, with respect to: **a** all topological scores of alignments with known ground truth node mapping, **b** all biological scores of alignments with known node mapping, **c** all topological scores of alignments with unknown node mapping, and **d** all biological scores of alignments with unknown node mapping.* Percentages* represent the scores achieved by an alignment quality measure.

Overall, the larger the neighborhood size, the better the alignment quality, even though all neighborhood sizes except T1 can in some cases result in higher-quality alignments than any other neighborhood size (Figure 9a, b). That is, for some values of network alignment parameters, smaller neighborhoods can produce higher-quality alignments than larger neighborhoods, which is a surprising though not alarming result. It is possible for larger neighborhood sizes to produce lower quality alignments due to nodes in one network having denser, more complex neighborhoods than nodes in the other network. For example, two nodes $u$ and $v$ from different networks can have similar neighborhoods at size e.g., T2 but different neighborhoods at larger size e.g., T3, if e.g., the 3-hop neighborhood of node $v$ in one network is empty while the 3-hop neighborhood of node $u$ in another network is not. Thus, although larger network neighborhoods include more of the network topological information, they could also “confuse” the network signal, depending on the topology of the aligned networks, in which case smaller neighborhoods may be preferred.

**Figure 10:**
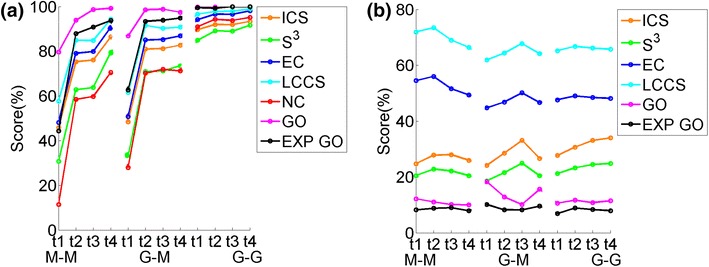
Detailed illustration of the effect of the neighborhood size for **a** synthetic and** b** real network data. In particular, results are shown for all three aligners, for the yeast-yeast 5% alignment at $\alpha =0.6$ in** a** and for the fly-worm alignment at $\alpha =0.4$ in** b**.* Percentages* represent the scores achieved by an alignment quality measure. For other network pairs and other values of $\alpha$, see Additional file 1: Figures S17–S21 and S22–S27.

When zooming into the results further to observe the effect of the aligner, the general trends from Figure 9a, b still hold independent of the aligner, but some fluctuations in the results exist (Additional file 1: Figures S17–S21). Namely, M-M generally prefers T3 and T4 neighborhood sizes. G-M prefers T2 in addition to T3 and T4, where T3 or T4 are actually inferior to T2 in some cases, depending on the noise level. G-G performs well on of T1-T4, with a slight preference of T3 or T4, depending on the noise level. See Figure 10a for an illustration.

When zooming into the results further to observe the effect of the $\alpha$ parameter, general trends from Figure 9a, b are overall the same for all values of $\alpha$ (Additional file 1: Figures S17–S21). The only exception is $\alpha =0$, which should not be used in the first place (“Summary”).

#### Real networks with unknown node mapping

Unlike for the synthetic networks, the largest neighborhood size (T4) is now not overly dominant over the smaller network sizes. Specifically, for real network data set, it is T3 that is the most dominant, followed by T4 and T2, which are tied, and followed by T1, which is inferior (Figure 9c, d).

When zooming into the results further to observe the effect of the aligner, we see that each aligner has an interesting behavior (Additional file 1: Figures S22–S27). Namely, M-M’s and G-G’s preference on the neighborhood size is mainly dictated by the choice of species whose networks are aligned. For G-M, in general, the larger neighborhood sizes are preferred; in some cases, depending on the species, G-M prefers T3 more than other neighborhood sizes. See Figure 10b for an illustration.

When zooming into the results further to observe the effect of the $\alpha$ parameter, just as for synthetic networks, the results from Figure 9c, d do not drastically change with the change of $\alpha$ value (Additional file 1: Figures S22–S27).

#### Summary

In general, the larger the neighborhood size within NCF, the higher the alignment quality. However, it is not necessarily the case that the largest neighborhood size always produces the best alignments nor that it is always dominant to the smaller neighborhood sizes. This means that slightly smaller neighborhood sizes (and T3 in particular) might be desirable, as this could not only produce better alignments in some cases but also decrease the computational complexity of the given method.

### Relationships between different alignment quality measures

We use a total of seven alignment quality measures: the ground truth NC measure that can only be measured in alignments of synthetic networks with known node mapping, four additional topological measures (EC, ICS, ${\rm S}^3$, and LCCS), and two biological measures (GO and EXP) (“Network alignment quality measures”). Here, we briefly comment on the relationship between the different measures.

**Figure 11:**
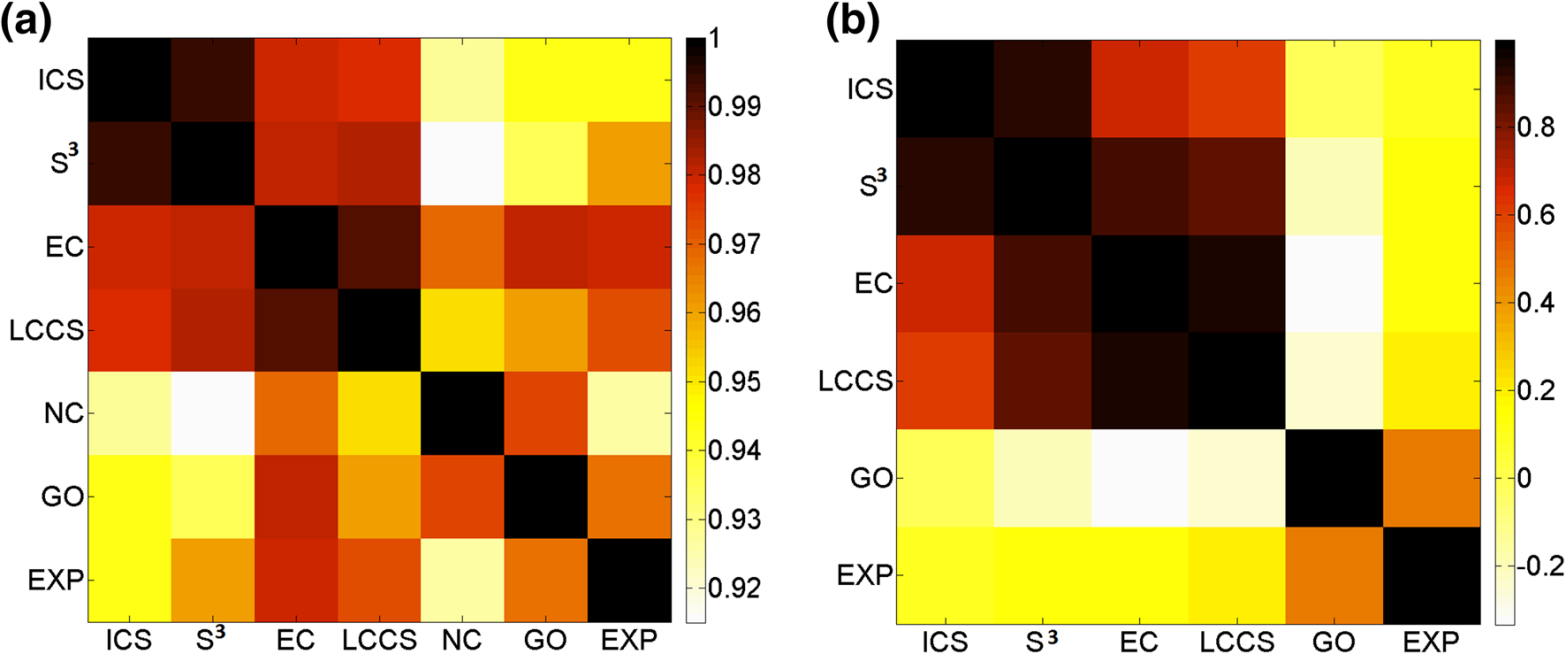
Pairwise correlations between different alignment quality measures. The results are shown for: **a** synthetic networks with known ground truth node mapping and **b** real networks with unknown node mapping. Correlations were computed over alignments with the highest NC scores in** a** and over alignments with the highest EC scores in** b** (because we do not known NC scores for alignments of real networks). Note that *color scales* for the two panels are different.

NC significantly correlates with both topological and biological alignment quality measures (Figure 11a), which is encouraging. Further, for the synthetic network data set, it is also encouraging that all other measures significantly correlate well (Pearson correlation coefficient of at least 0.8), even though we see some clustering of the topological measures and also of the biological measures (Figure 11a). Interestingly, each of the two biological measures, GO and EXP, correlates better with some of the topological measures (e.g., EC) than with each other.

Unlike for the synthetic network data, for the real network data, the topological measures now correlate poorly with the biological measures (Pearson correlation coefficient of at most 0.2; Figure 11b). Importantly, this implies that for the real network data set it might be hard to produce an alignment that is of excellent quality both topologically and biologically. Also, while we again see clustering of the topological measures, the two biological measures now correlate weakly (Figure 11b), indicating that the choice of GO annotation data obtained by experimental evidence code matters (“Network alignment quality measures”).

The result differences between the synthetic networks and the real networks could be due to differences in their properties (“Data sets”).

Note that when measuring the correlations between the different alignment quality measures, we have used the Pearson correlation coefficient. In case that the data is not necessarily normally distributed, using a non-parametric (i.e., distribution-free) measure of correlation would be appropriate. Hence, we repeat the above analysis with respect to such a measure, namely the Spearman correlation coefficient. Importantly, our results produced in this way are mostly consistent to the results produced when using the Pearson correlation coefficient (Additional file 1: Figure S28).

## Conclusions

We have aimed to systematically answer three questions in the context of MI-GRAAL and GHOST network aligners: (1) what is the contribution of each method’s NCF and AS to the alignment quality, (2) how much sequence versus topology information should be used within NCF when generating an alignment, and (3) how large the size of the neighborhoods of the compared nodes from different networks should be. Our results show that: (1) MI-GRAAL’s NCF is superior to GHOST’s, while the performance of their ASs is data-dependent, (2) some amount of topological data should be used in the NCF, and (3) the larger the amount of topology, the better, although using the second largest neighborhood size can result in better results and lower computational complexity compared to using the largest neighborhood size. Our results represent a set of general recommendations for a fair evaluation of any GNA method (and especially if the method falls into the two-state NCF-AS category), not just MI-GRAAL and GHOST.

Genomic sequence alignment has revolutionized our biomedical understanding. Biological network alignment has already had similar impacts. And given the tremendous amounts of biological network data that continue to be produced, network alignment will only continue to gain importance. The hope is that it could lead to new discoveries about the principles of life, evolution, disease, and therapeutics. Network alignment has also strived in other domains as well, with applications such as semantically matching entities in different ontologies [8] or comparing online social networks with impacts on user privacy [9].

## Additional files

Additional file 1: Supplementary material containing additional results.

## Abbreviations

GNA: global network aligner
LNA: local network aligner
NCF: node cost function
AS: alignment Strategy
PPI: protein–protein interaction
GDV: graphlet degree vector.
