# Supplementary material for: Fair evaluation of global network aligners
Source: Algorithms Mol Biol. 2015 Jun 9;10:19. doi: 10.1186/s13015-015-0050-8 (PMC4460690; doi:10.1186/s13015-015-0050-8)
Supplement: Supplementary file 1 — Supplementary material containing additional results. [file 13015_2015_50_MOESM1_ESM.pdf]

SUPPLEMENTARY MATERIAL FOR: FAIR EVALUATION OF GLOBAL NETWORK ALIGNERS

JOSEPH CRAWFORD, YIHAN SUN, TIJANA MILENKOVIĆ

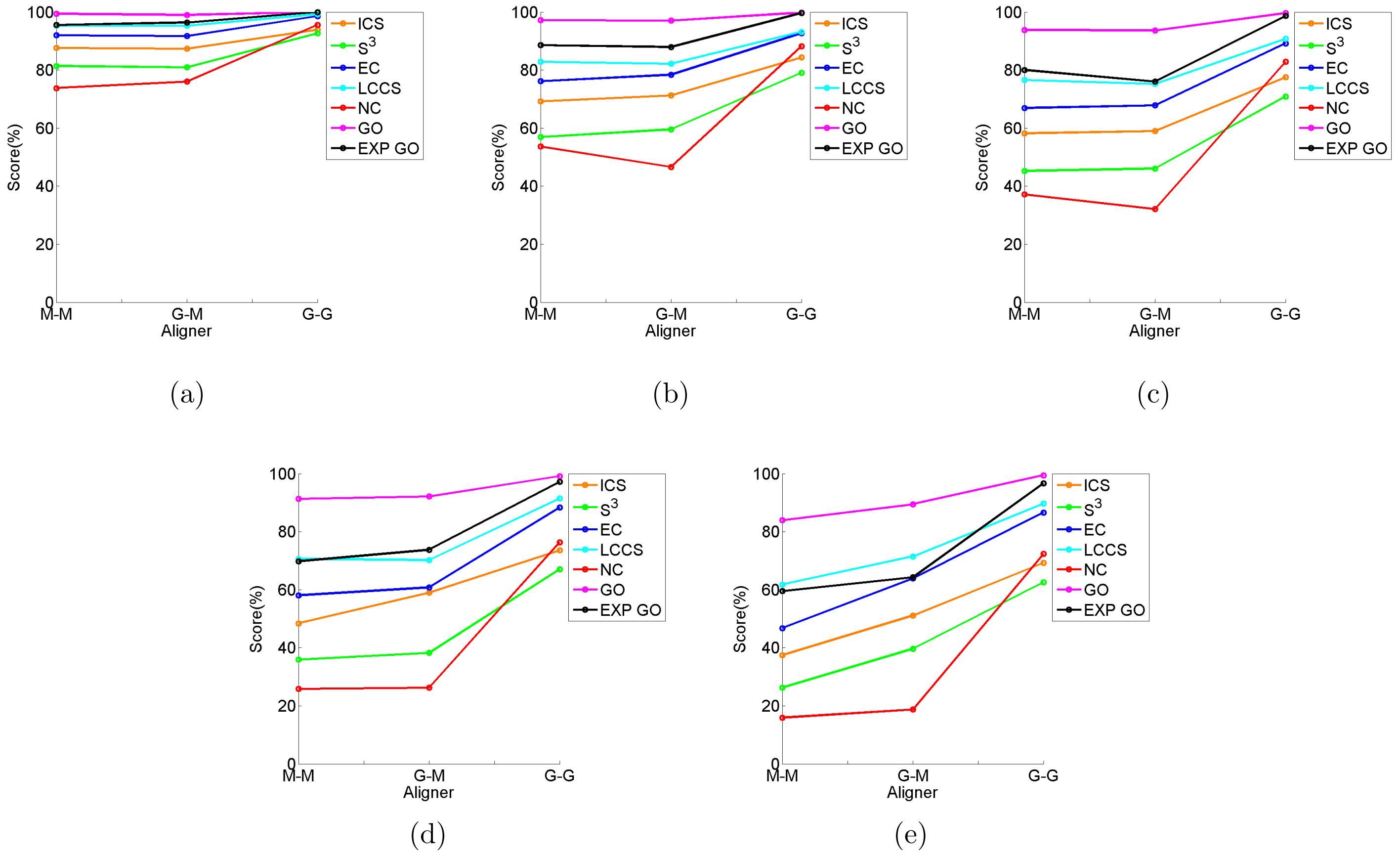

Supplementary Figure 1. Alignment quality results of the three aligners (M-M, G-M, and G-G) for *best* alignments over all values of α and all neighborhood sizes with respect to each of the five topological, and two biological alignment quality measures. Network pairs by panel: **(a)** Yeast-Yeast 5%, **(b)** Yeast-Yeast 10%,**(c)** Yeast-Yeast 15%,**(d)** Yeast-Yeast 20%,**(e)** Yeast-Yeast 25%.

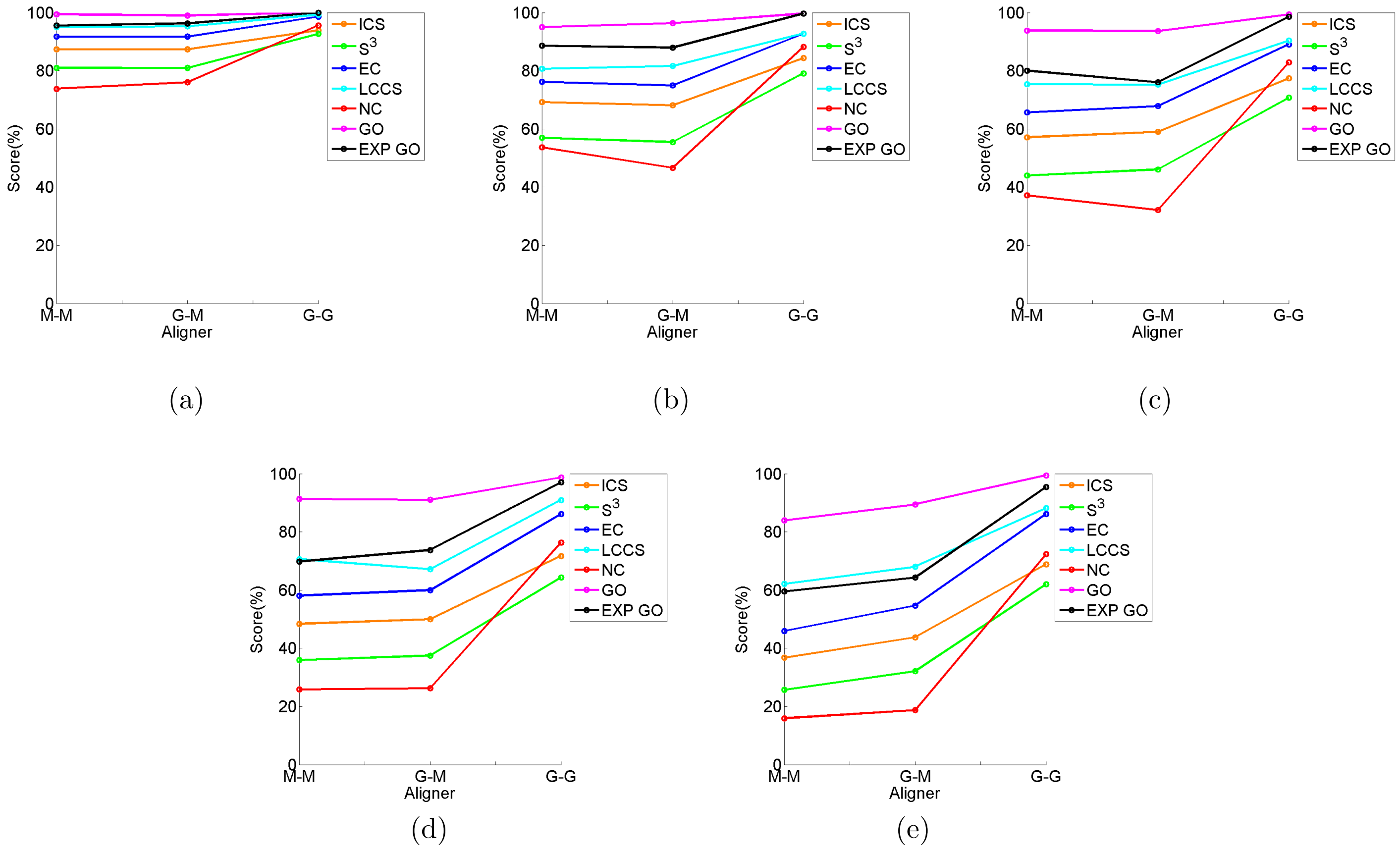

Supplementary Figure 2. Alignment quality results of the three aligners (M-M, G-M, and G-G) where the five topological and two biological alignment quality scores are taken from the alignment with the highest node correctness score. Network pairs by panel: **(a)** Yeast-Yeast 5%, **(b)** Yeast-Yeast 10%,**(c)** Yeast-Yeast 15%,**(d)** Yeast-Yeast 20%,**(e)** Yeast-Yeast 25%.

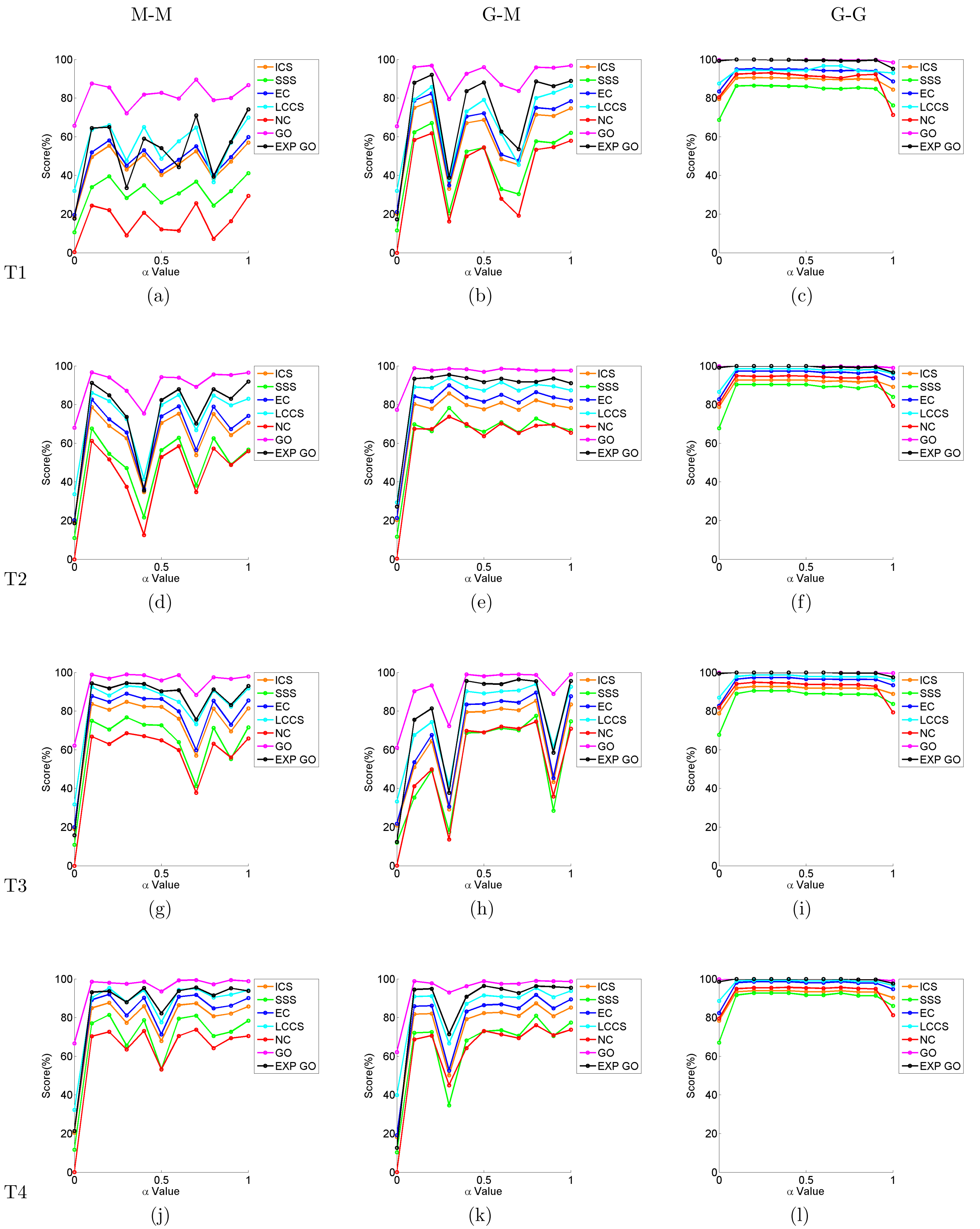

Supplementary Figure 3. Detailed illustration of the effect of the α parameter on the five topological and two biological alignment quality scores for the three aligners (M-M, G-M, G-G) for the yeast-yeast 5% alignments. Each row represents a different neighborhood size (T1, T2, T3, T4)

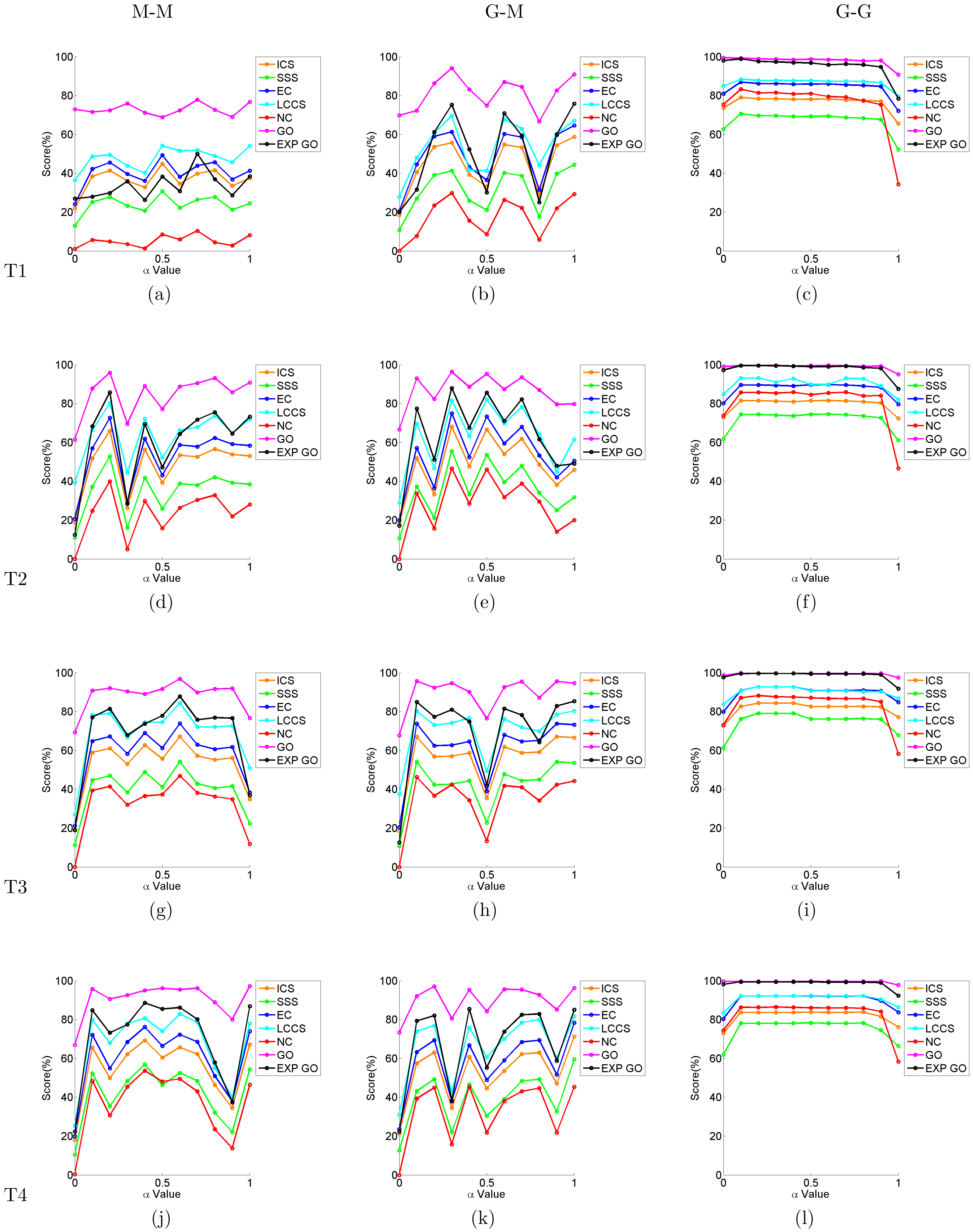

Supplementary Figure 4. Quality of noisy yeast alignments, for 10% noise, with respect to raw scores as a function of α.

M-M G-M G-G

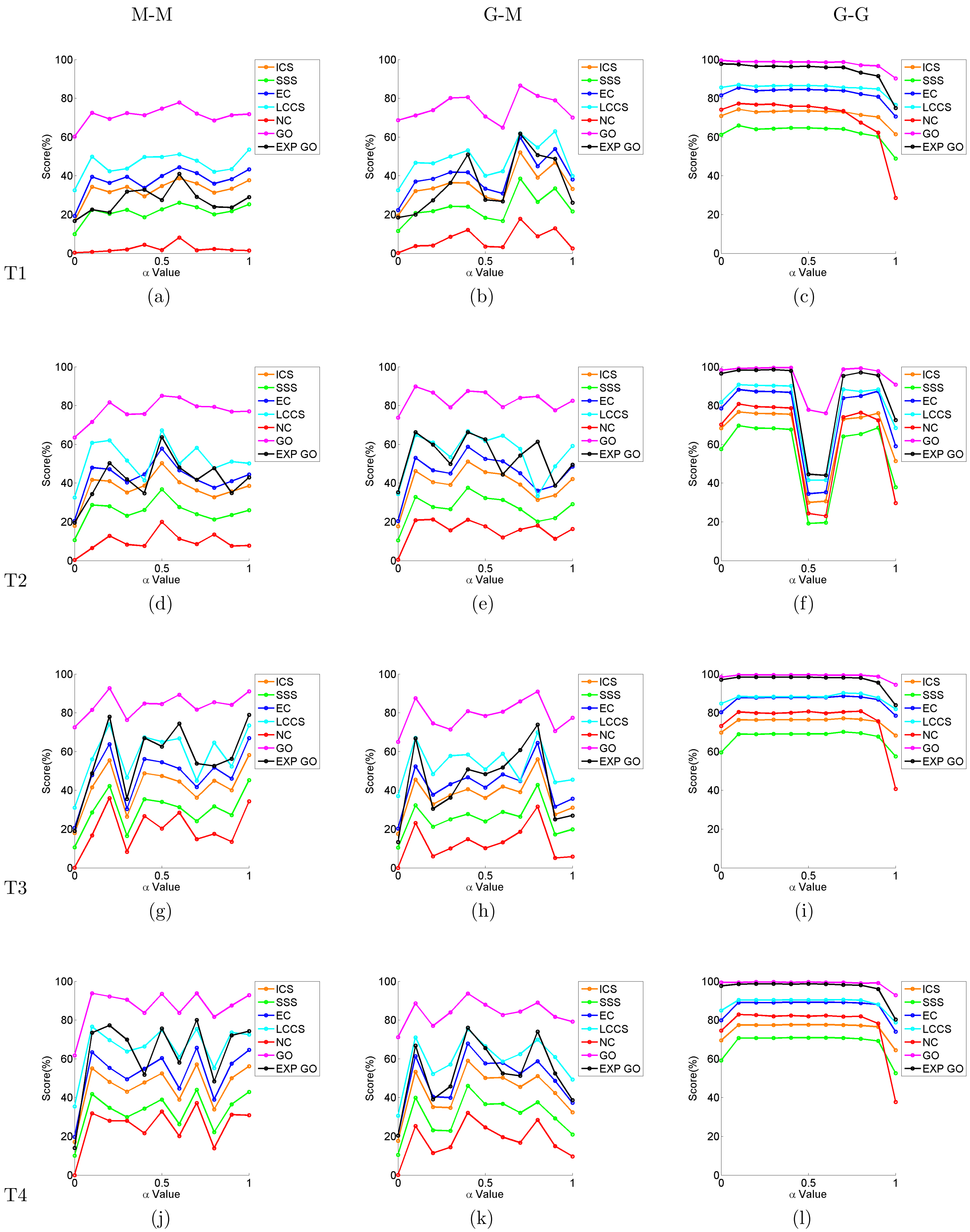

SUPPLEMENTARY FIGURE 5. Quality of noisy yeast alignments, for 15% noise, with respect to <u>raw</u> scores as a function of α.

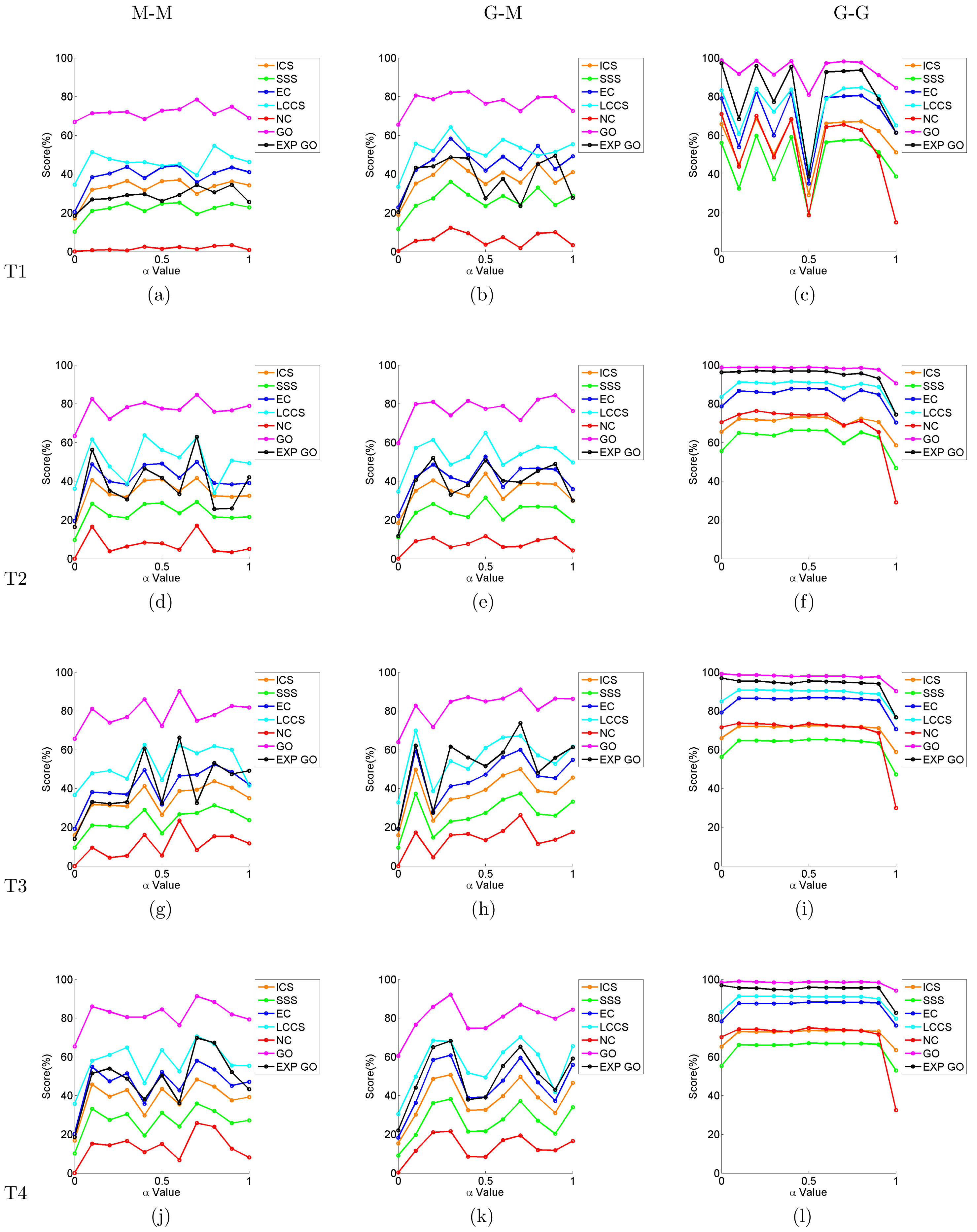

Supplementary Figure 6. Quality of noisy yeast alignments, for 20% noise, with respect to raw scores as a function of α.

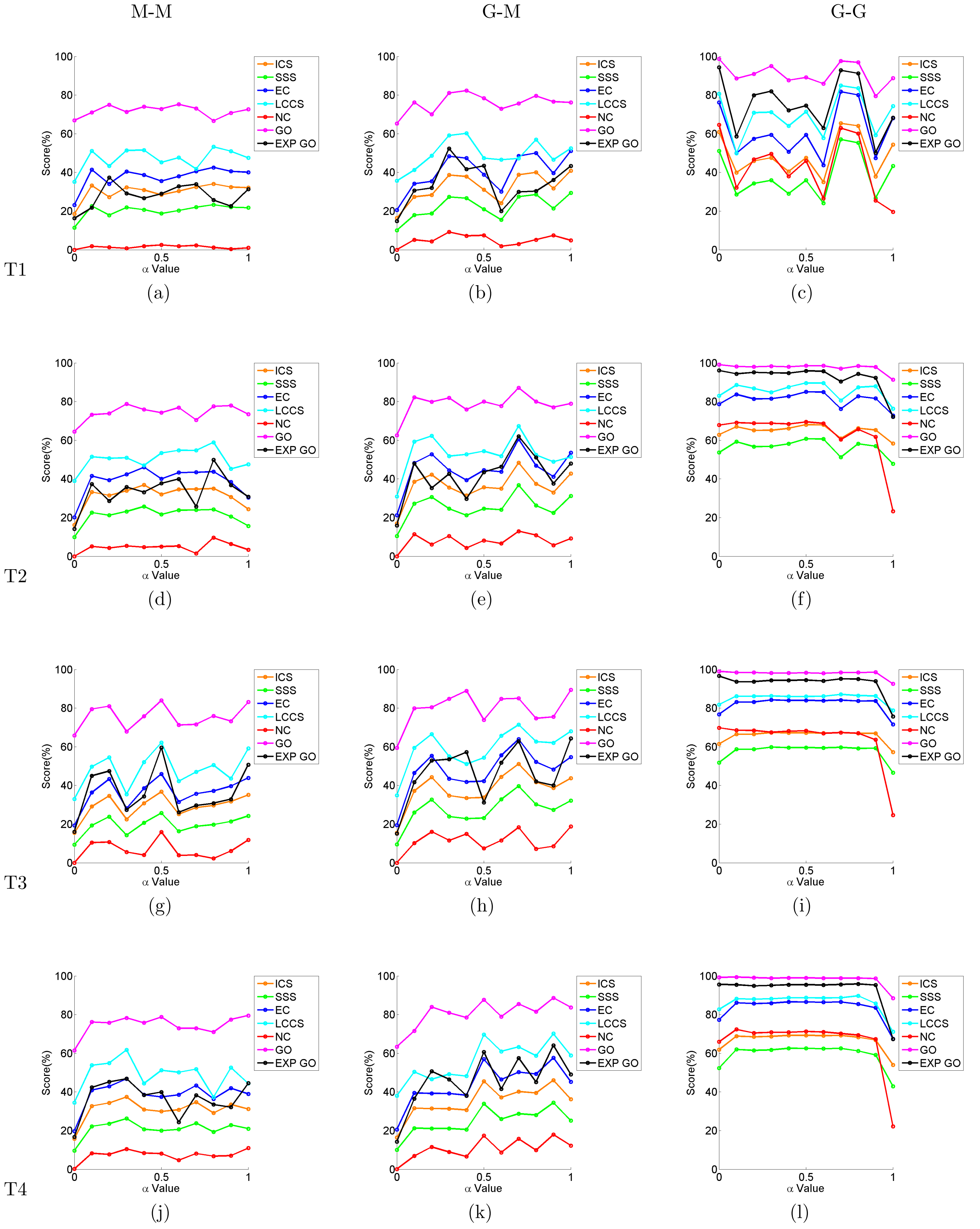

Supplementary Figure 7. Quality of noisy yeast alignments, for 25% noise, with respect to <u>raw</u> scores as a function of α.

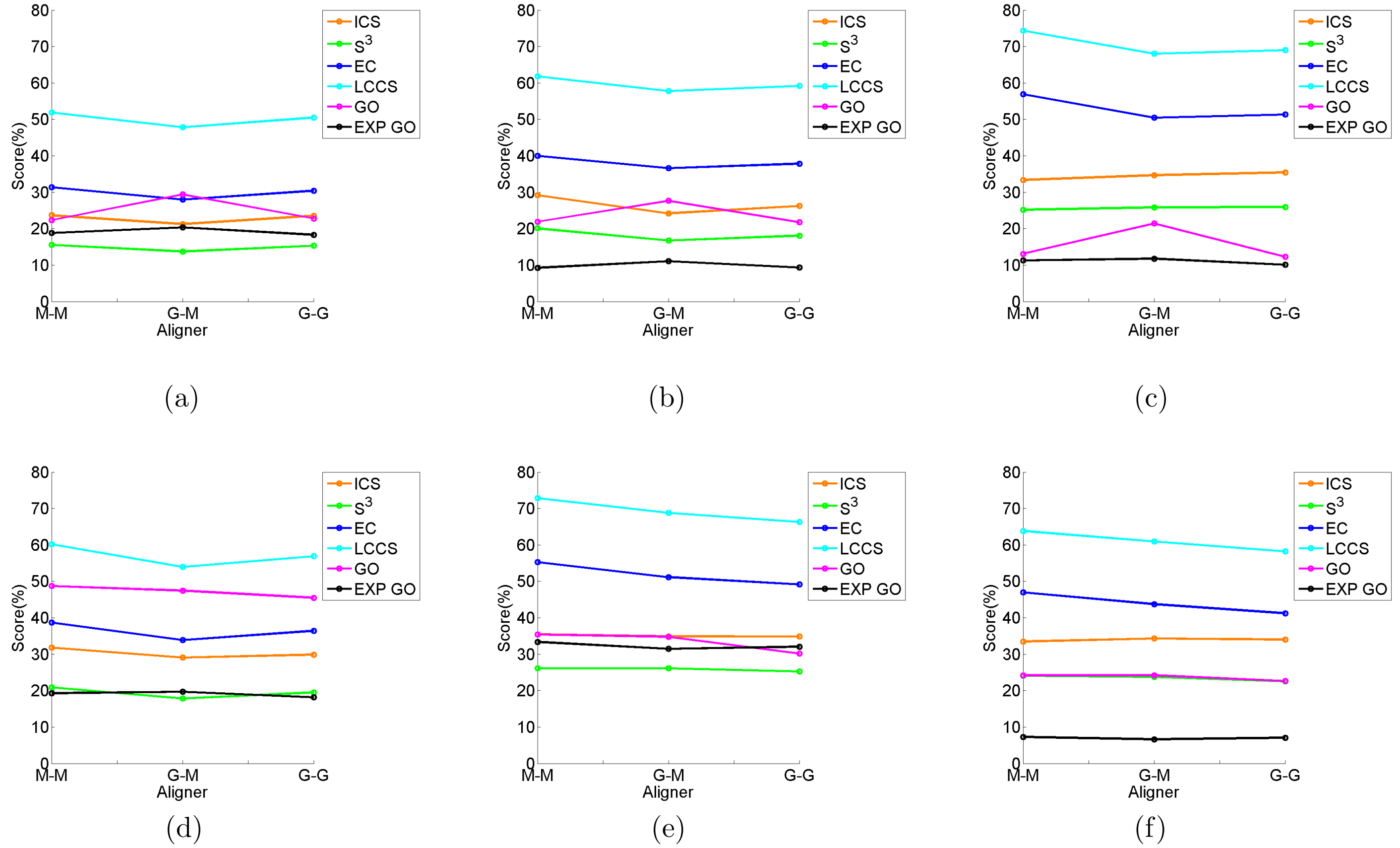

Supplementary Figure 8. Alignment quality results of the three aligners (M-M, G-M, and G-G) for *best* alignments over all values of α and all neighborhood sizes with respect to each of the four topological, and two biological alignment quality measures. Network pairs by panel: **(a)** Fly-Human, **(b)** Fly-Yeast,**(c)** Fly-Worm,**(d)** Human-Yeast,**(e)** Human-Worm,**(f)** Worm-Yeast.

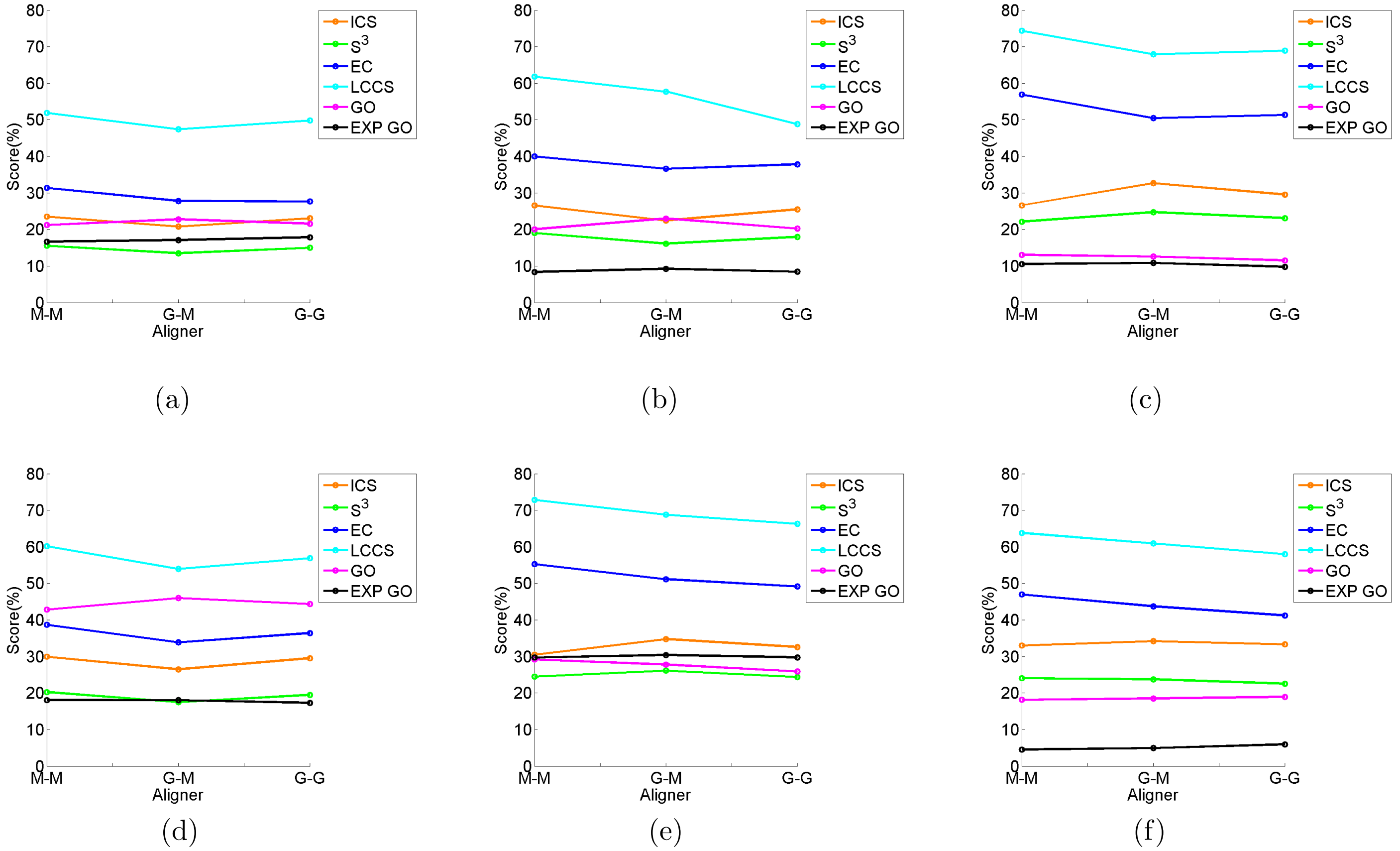

Supplementary Figure 9. Alignment quality results of the three aligners (M-M, G-M, and G-G) where the four topological and two biological alignment quality scores are taken from the alignment with the highest edge correctness score. Network pairs by panel: **(a)** Fly-Human, **(b)** Fly-Yeast,**(c)** Fly-Worm,**(d)** Human-Yeast,**(e)** Human-Worm,**(f)** Worm-Yeast.

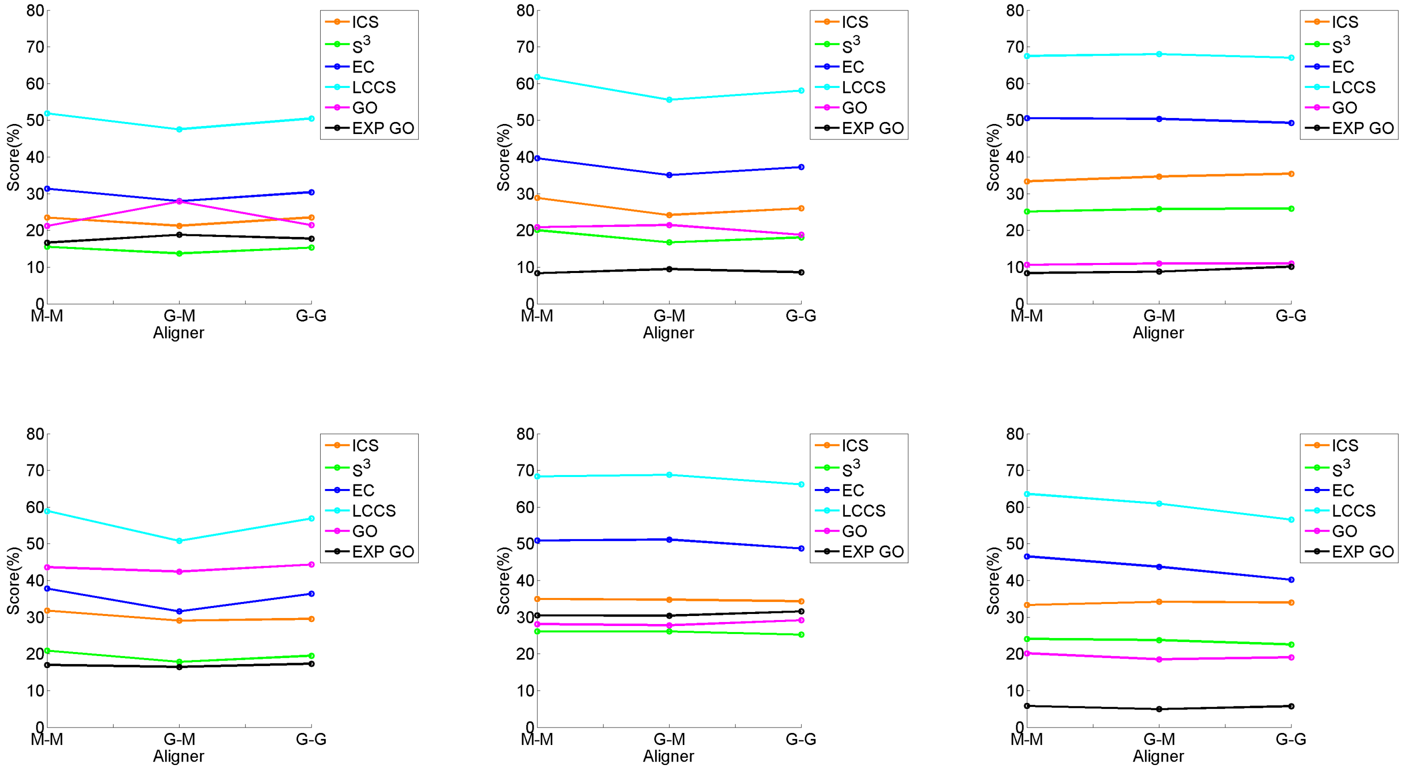

Supplementary Figure 10. Alignment quality results of the three aligners (M-M, G-M, and G-G) where the four topological and two biological alignment quality scores are taken from the alignment with the highest symmetric substructure score. Network pairs by panel: **(a)** Fly-Human, **(b)** Fly-Yeast,**(c)** Fly-Worm,**(d)** Human-Yeast,**(e)** Human-Worm,**(f)** Worm-Yeast.

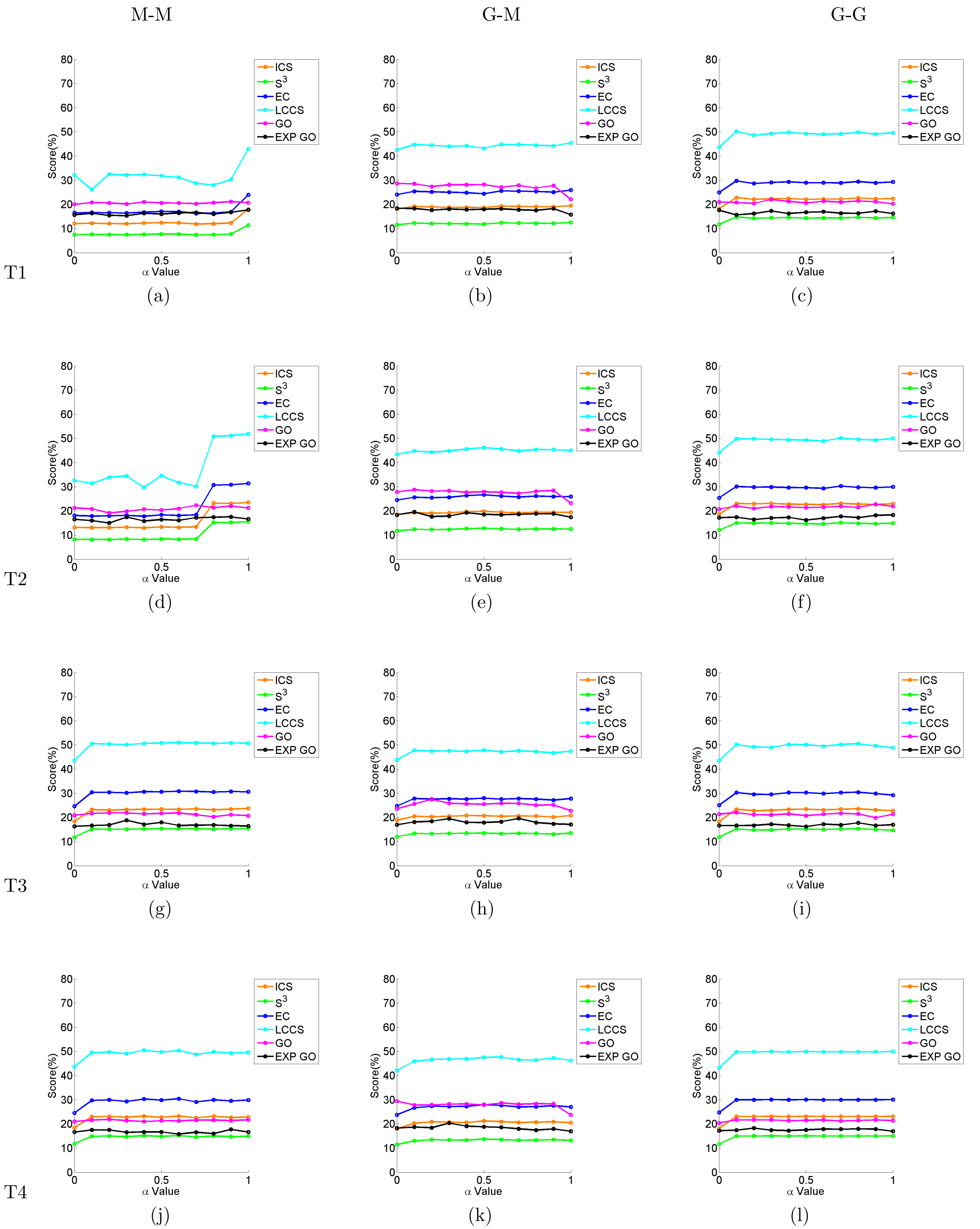

Supplementary Figure 11. Detailed illustration of the effect of the α parameter on the five topological and two biological alignment quality scores for the three aligners (M-M, G-M, G-G) for the fly-human alignments. Each row represents a different neighborhood size (T1, T2, T3, T4)

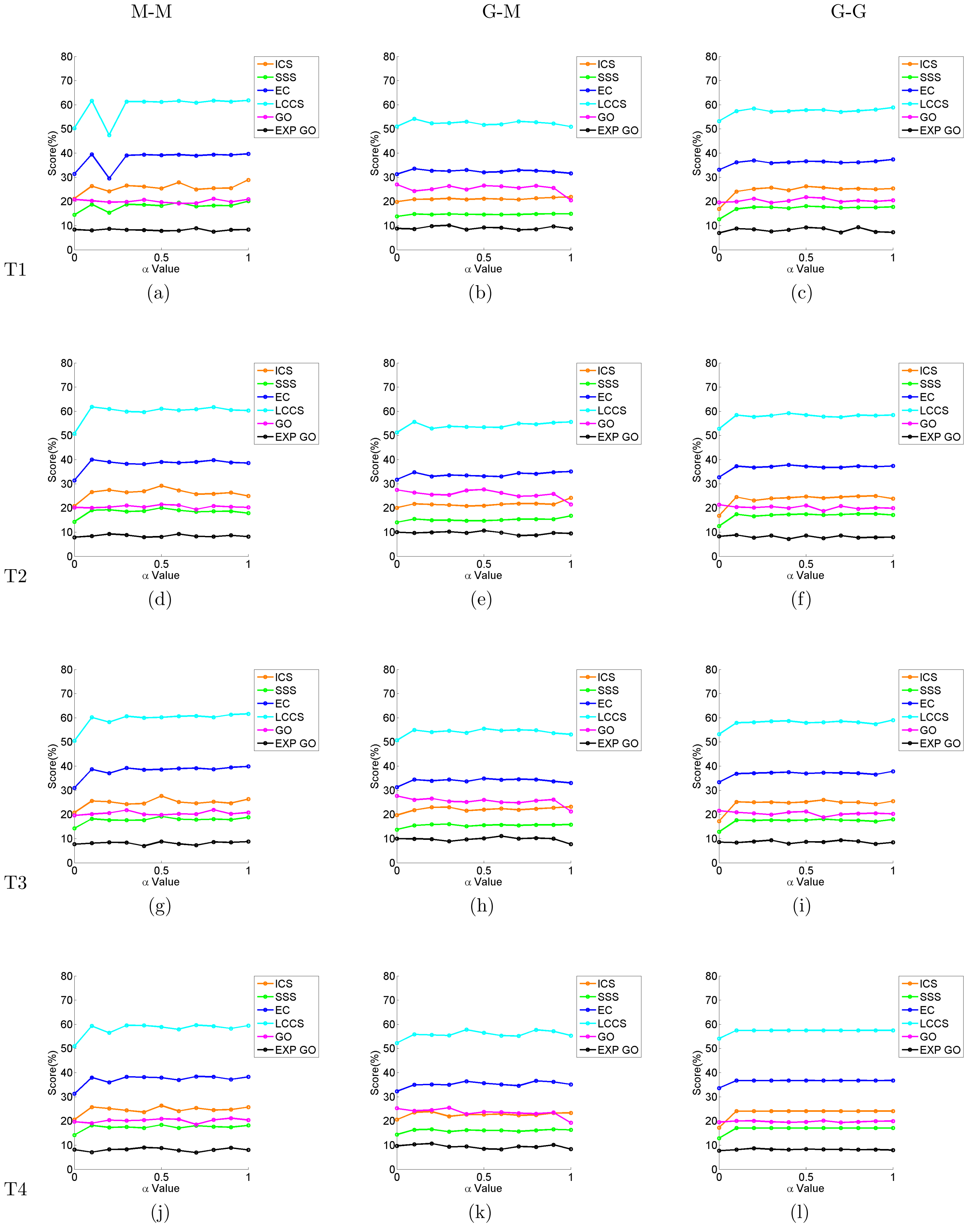

Supplementary Figure 12. Quality of Fly-Yeast alignments, with respect to raw scores as a function of α.

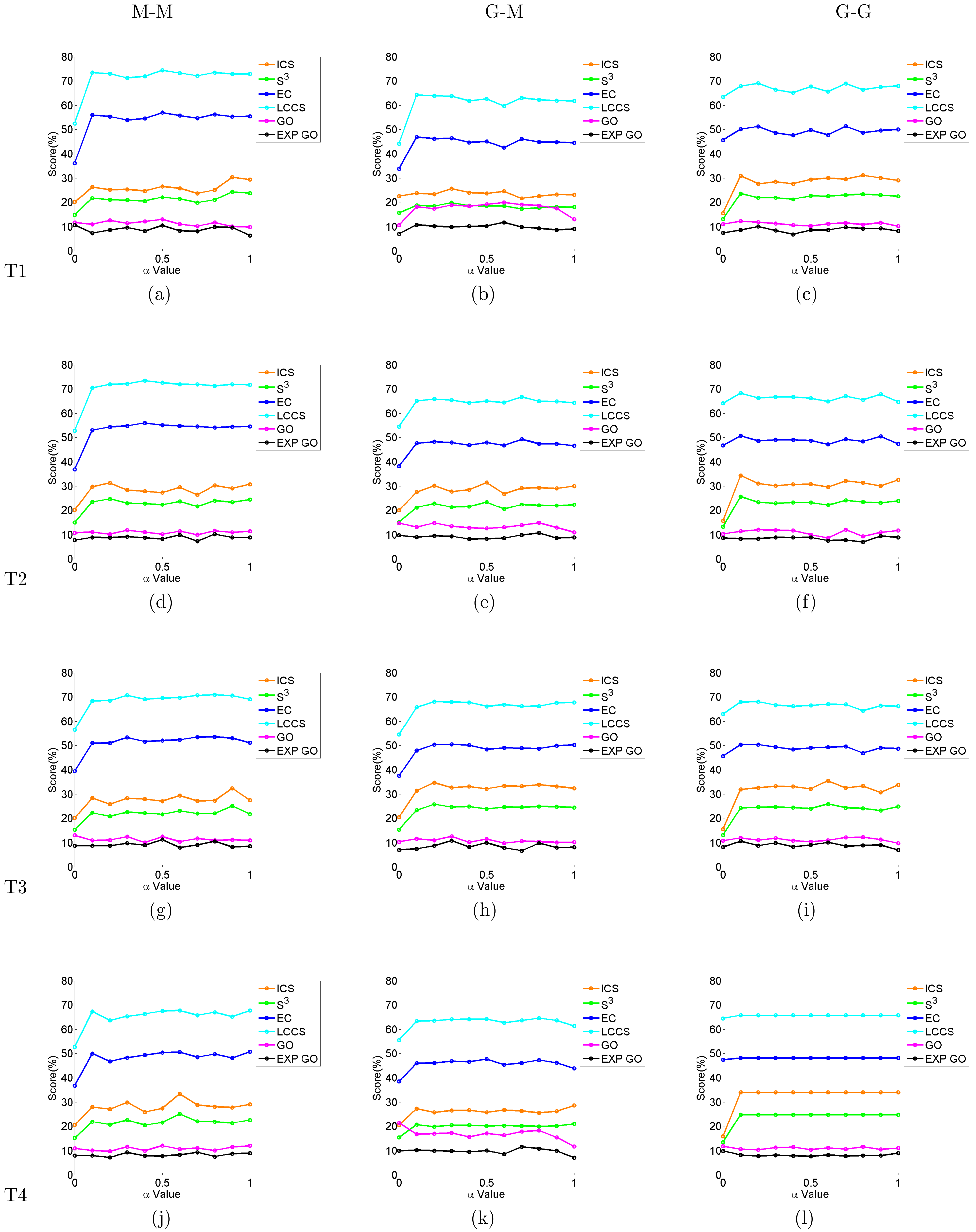

Supplementary Figure 13. Quality of Fly-Worm alignments, with respect to <u>raw</u> scores as a function of α.

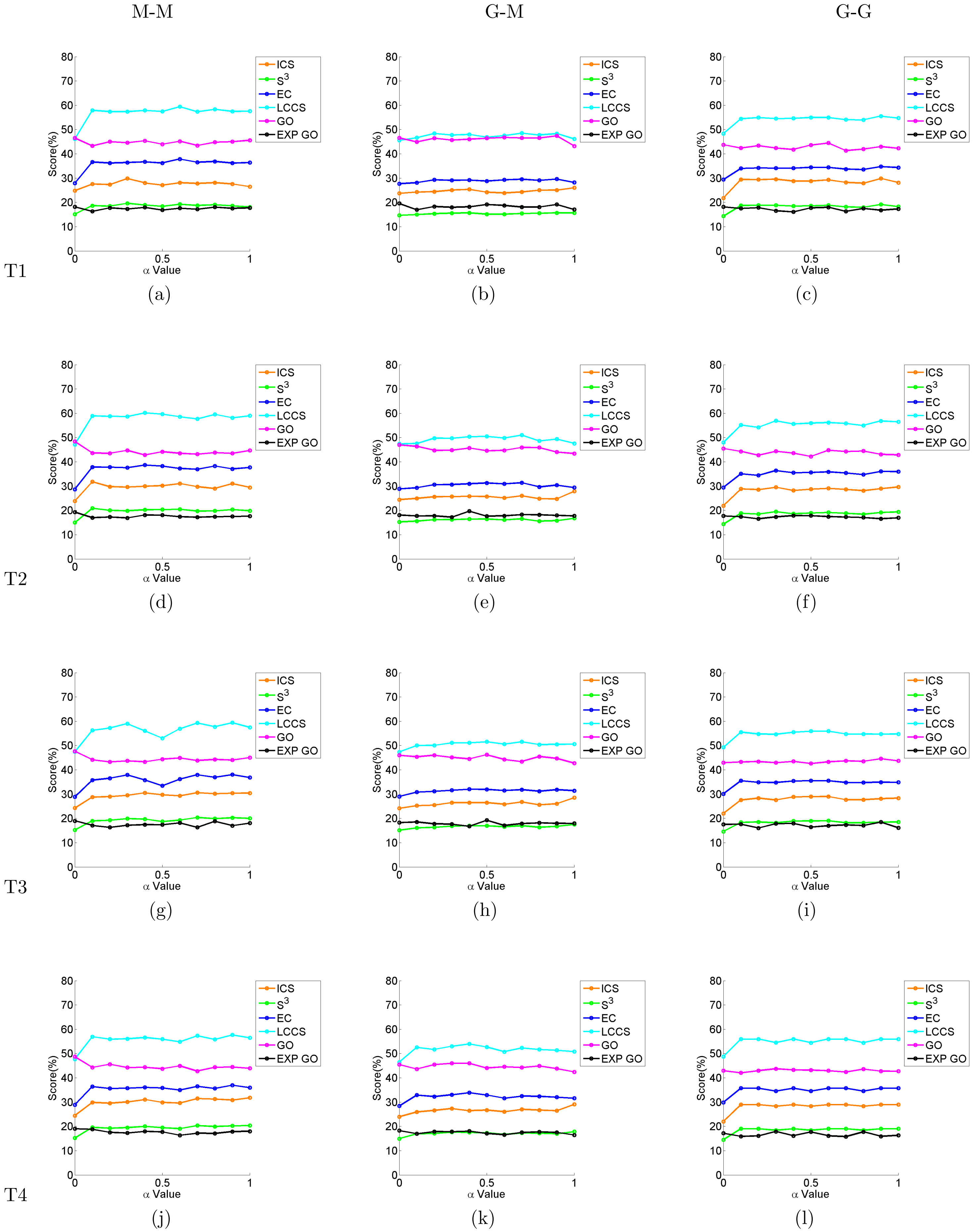

Supplementary Figure 14. Quality of Human-Yeast alignments, with respect to raw scores as a function of α.

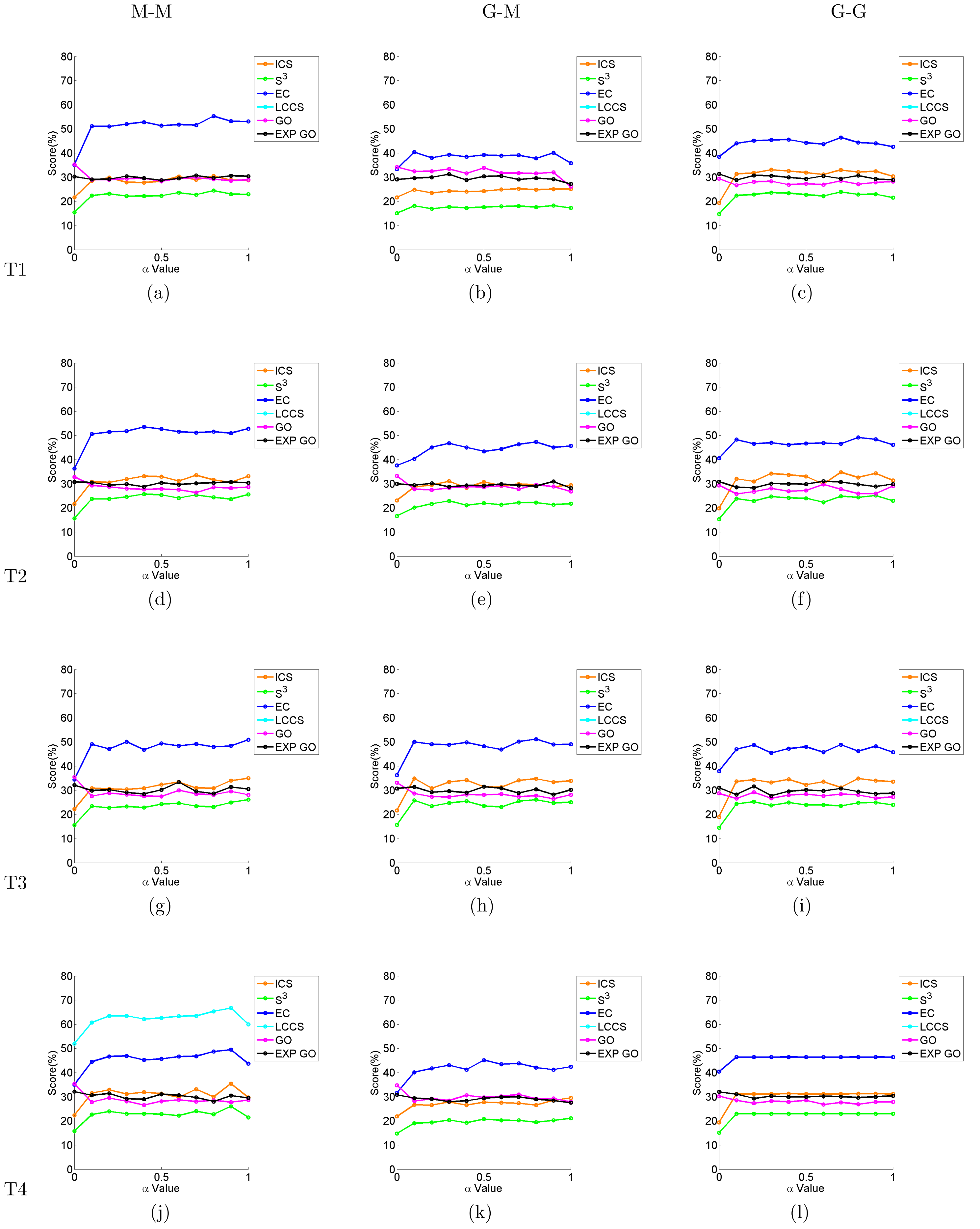

Supplementary Figure 15. Quality of Human-Worm alignments, with respect to <u>raw</u> scores as a function of α

.

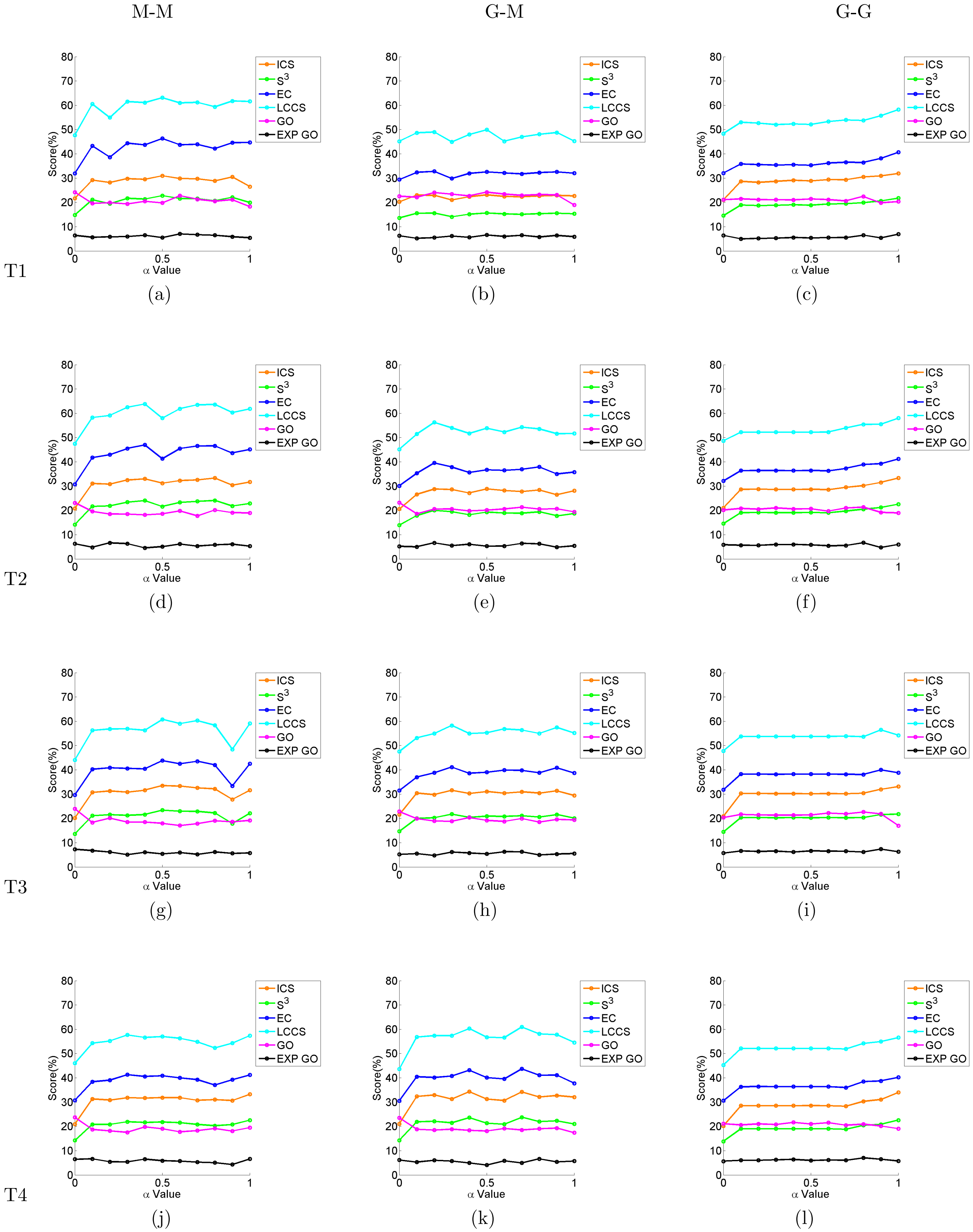

Supplementary Figure 16. Quality of Worm-Yeast alignments, with respect to raw scores as a function of α.

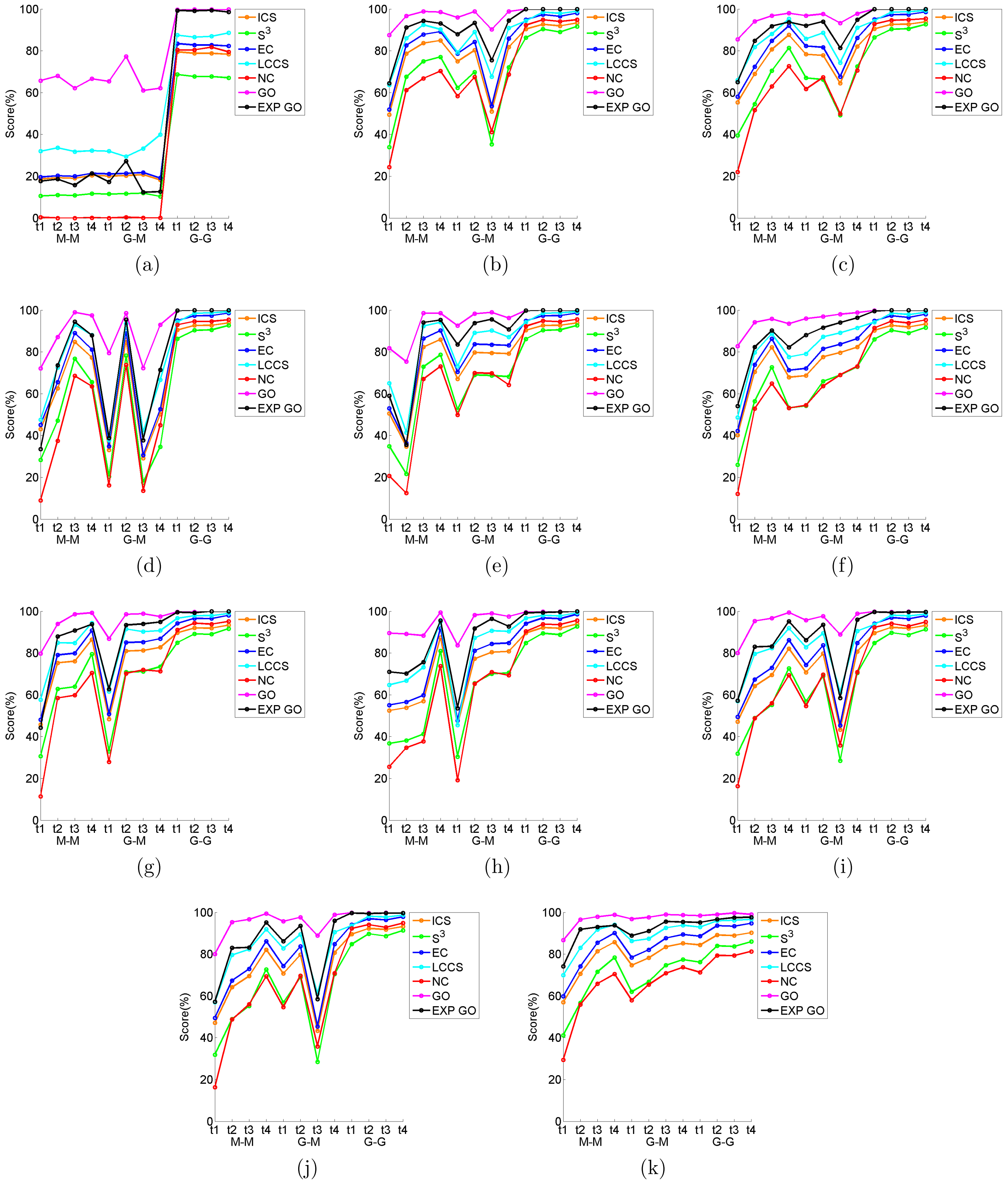

Supplementary Figure 17. Detailed illustration of the effect of the neighborhood size on each of the five topological and two biological alignment quality measures on each aligner (M-M, G-M, G-G) for the yeast-yeast 5% alignments. Values of α by panel: **(a)** $\alpha = 0.0$, **(b)** $\alpha = 0.1$,**(c)** $\alpha = 0.2$,**(d)** $\alpha = 0.3$,**(e)** $\alpha = 0.4$,**(f)** $\alpha = 0.5$,**(g)** $\alpha = 0.6$,**(h)** $\alpha = 0.7$,**(i)** $\alpha = 0.8$,**(j)** $\alpha = 0.9$,**(k)** $\alpha = 1.0$.

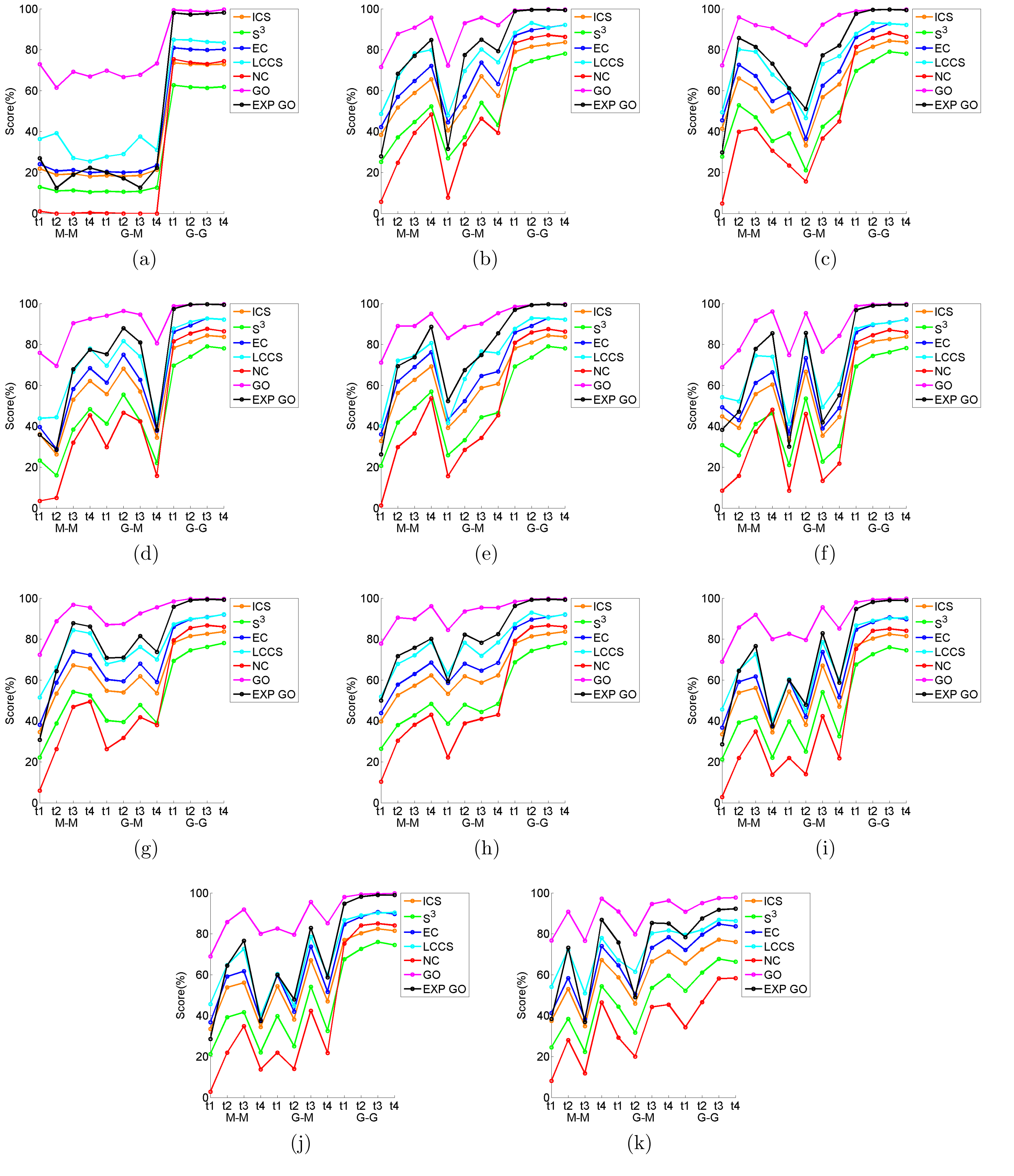

Supplementary Figure 18. Quality of noisy yeast alignments, for 10% noise, with respect to the highest raw scores as a function of the aligner. Values of α by panel: **(a)** $\alpha = 0.0$, **(b)** $\alpha = 0.1$,**(c)** $\alpha = 0.2$,**(d)** $\alpha = 0.3$,**(e)** $\alpha = 0.4$,**(f)** $\alpha = 0.5$,**(g)** $\alpha = 0.6$,**(h)** $\alpha = 0.7$,**(i)** $\alpha = 0.8$,**(j)** $\alpha = 0.9$,**(k)** $\alpha = 1.0$.

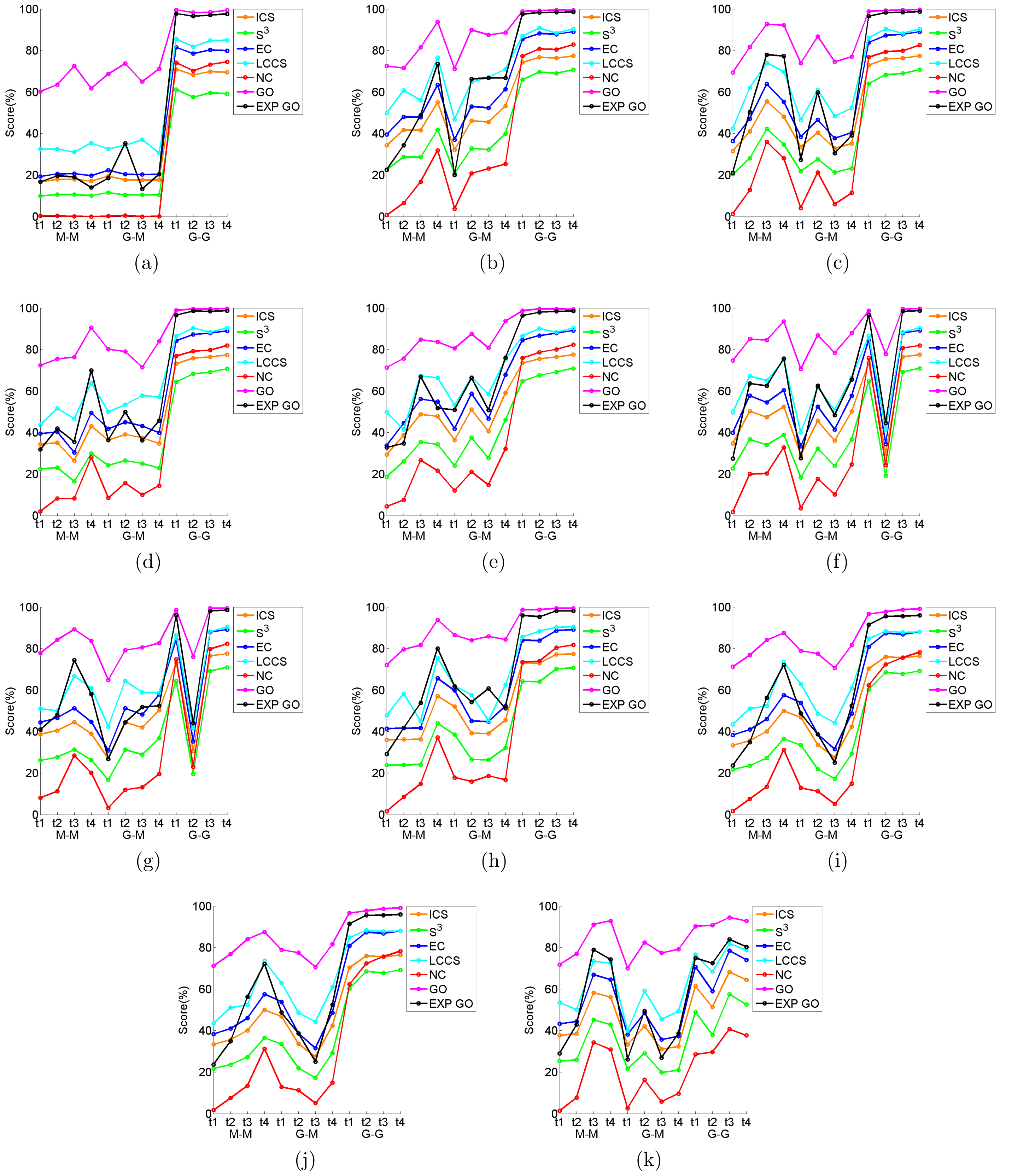

Supplementary Figure 19. Quality of noisy yeast alignments, for 15% noise, with respect to the highest raw scores as a function of the aligner. Values of α by panel: **(a)** $\alpha = 0.0$, **(b)** $\alpha = 0.1$,**(c)** $\alpha = 0.2$,**(d)** $\alpha = 0.3$,**(e)** $\alpha = 0.4$,**(f)** $\alpha = 0.5$,**(g)** $\alpha = 0.6$,**(h)** $\alpha = 0.7$,**(i)** $\alpha = 0.8$,**(j)** $\alpha = 0.9$,**(k)** $\alpha = 1.0$.

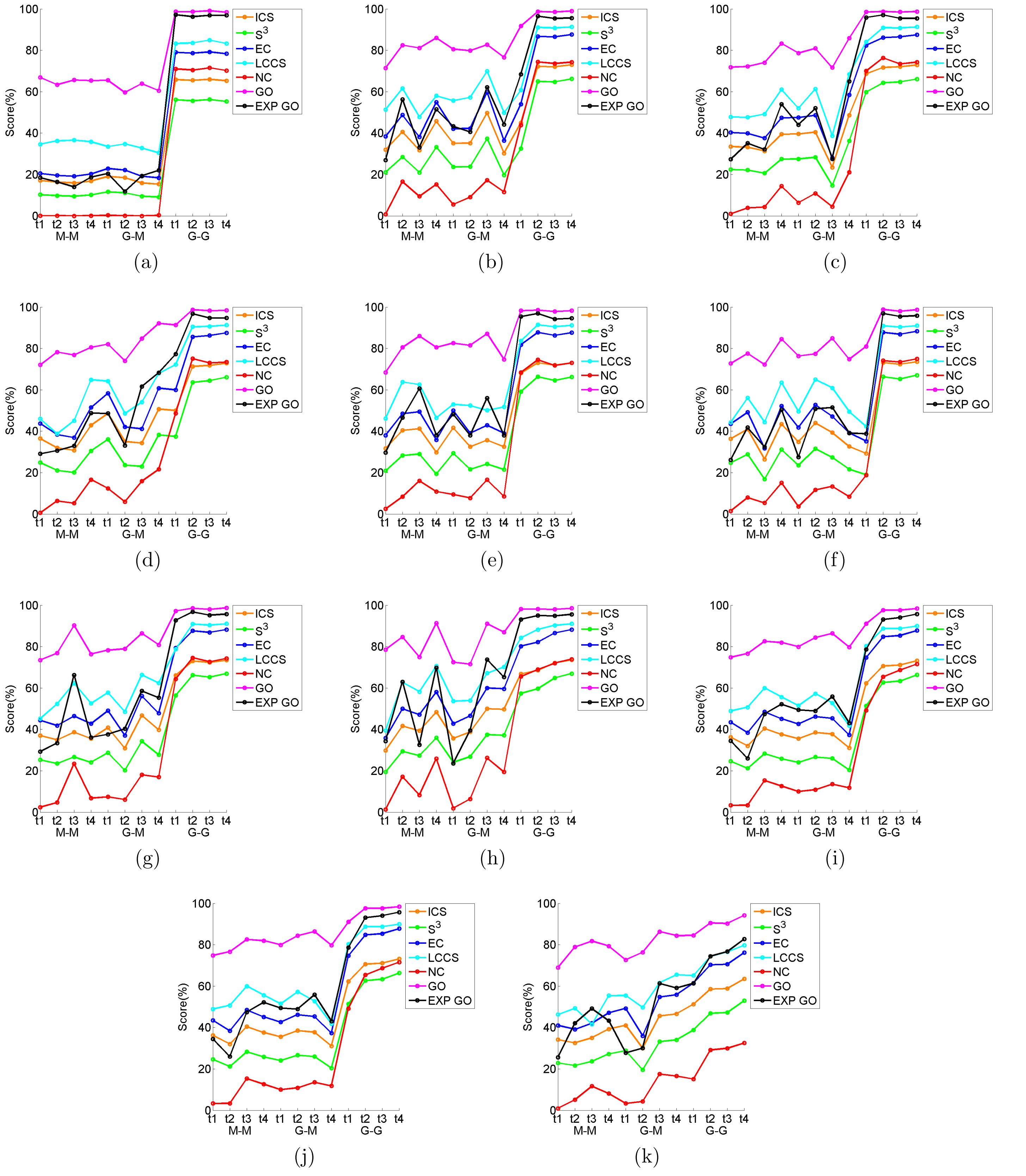

SUPPLEMENTARY FIGURE 20. Quality of noisy yeast alignments, for 20% noise, with respect to the highest raw scores as a function of the aligner. Values of α by panel: **(a)** $\alpha = 0.0$, **(b)** $\alpha = 0.1$,**(c)** $\alpha = 0.2$,**(d)** $\alpha = 0.3$,**(e)** $\alpha = 0.4$,**(f)** $\alpha = 0.5$,**(g)** $\alpha = 0.6$,**(h)** $\alpha = 0.7$,**(i)** $\alpha = 0.8$,**(j)** $\alpha = 0.9$,**(k)** $\alpha = 1.0$.

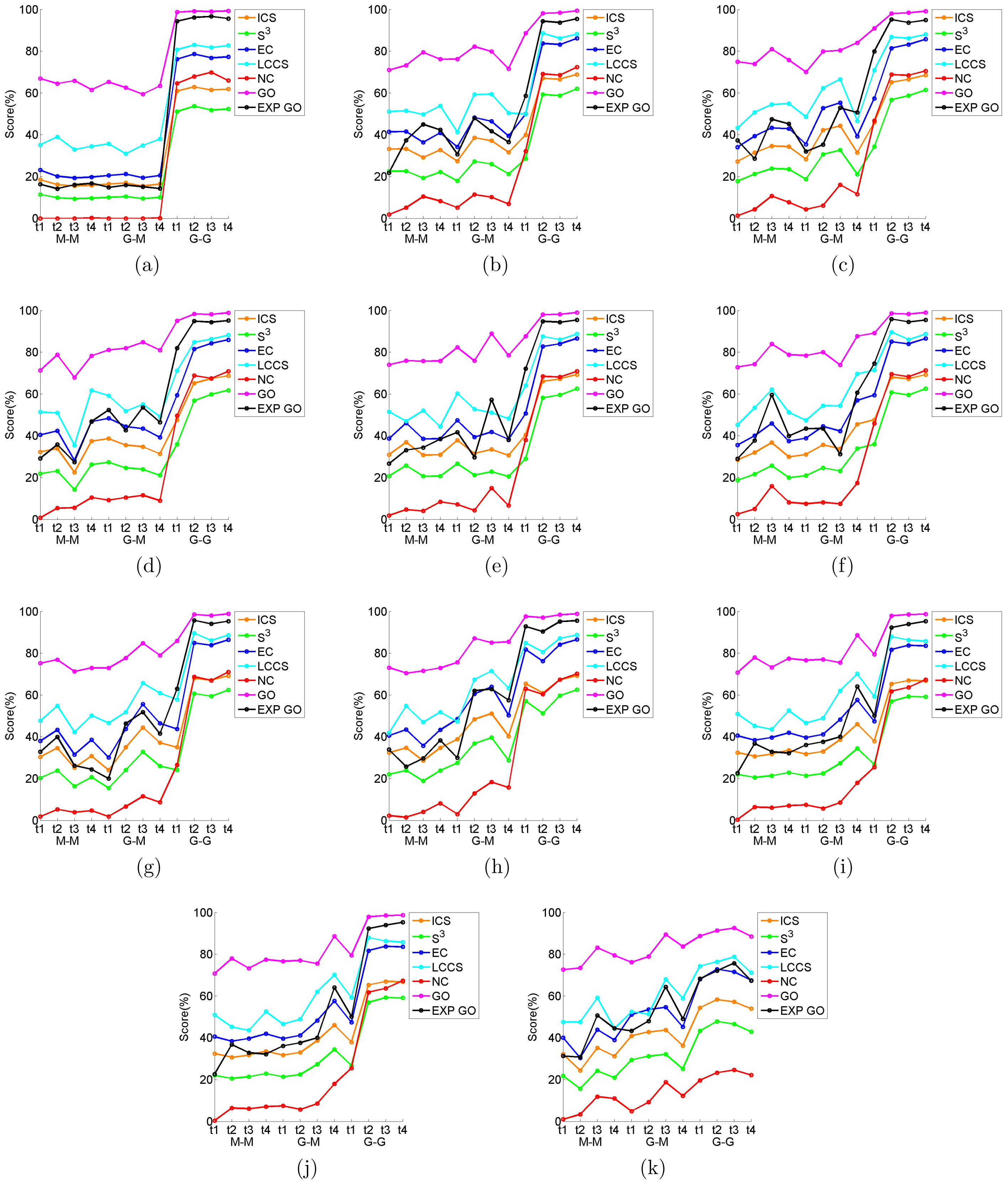

Supplementary Figure 21. Quality of noisy yeast alignments, for 25% noise, with respect to the highest raw scores as a function of the aligner. Values of α by panel: **(a)** $\alpha = 0.0$, **(b)** $\alpha = 0.1$,**(c)** $\alpha = 0.2$,**(d)** $\alpha = 0.3$,**(e)** $\alpha = 0.4$,**(f)** $\alpha = 0.5$,**(g)** $\alpha = 0.6$,**(h)** $\alpha = 0.7$,**(i)** $\alpha = 0.8$,**(j)** $\alpha = 0.9$,**(k)** $\alpha = 1.0$.

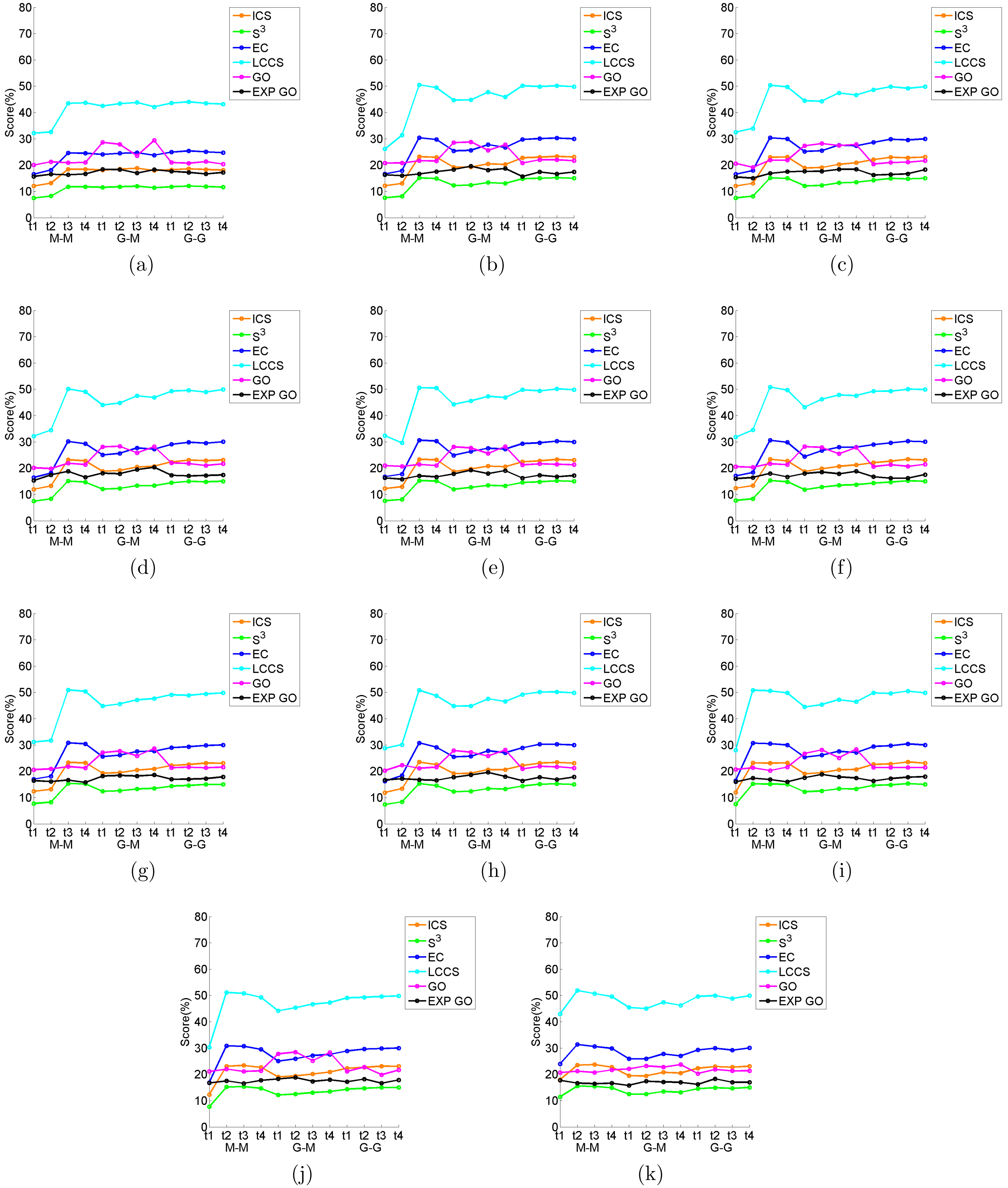

Supplementary Figure 22. Detailed illustration of the effect of the neighborhood size on each of the four topological and two biological alignment quality measures on each aligner (M-M, G-M, G-G) for the fly-human alignments. Values of α by panel: **(a)** $\alpha = 0.0$, **(b)** $\alpha = 0.1$,**(c)** $\alpha = 0.2$,**(d)** $\alpha = 0.3$,**(e)** $\alpha = 0.4$,**(f)** $\alpha = 0.5$,**(g)** $\alpha = 0.6$,**(h)** $\alpha = 0.7$,**(i)** $\alpha = 0.8$,**(j)** $\alpha = 0.9$,**(k)** $\alpha = 1.0$.

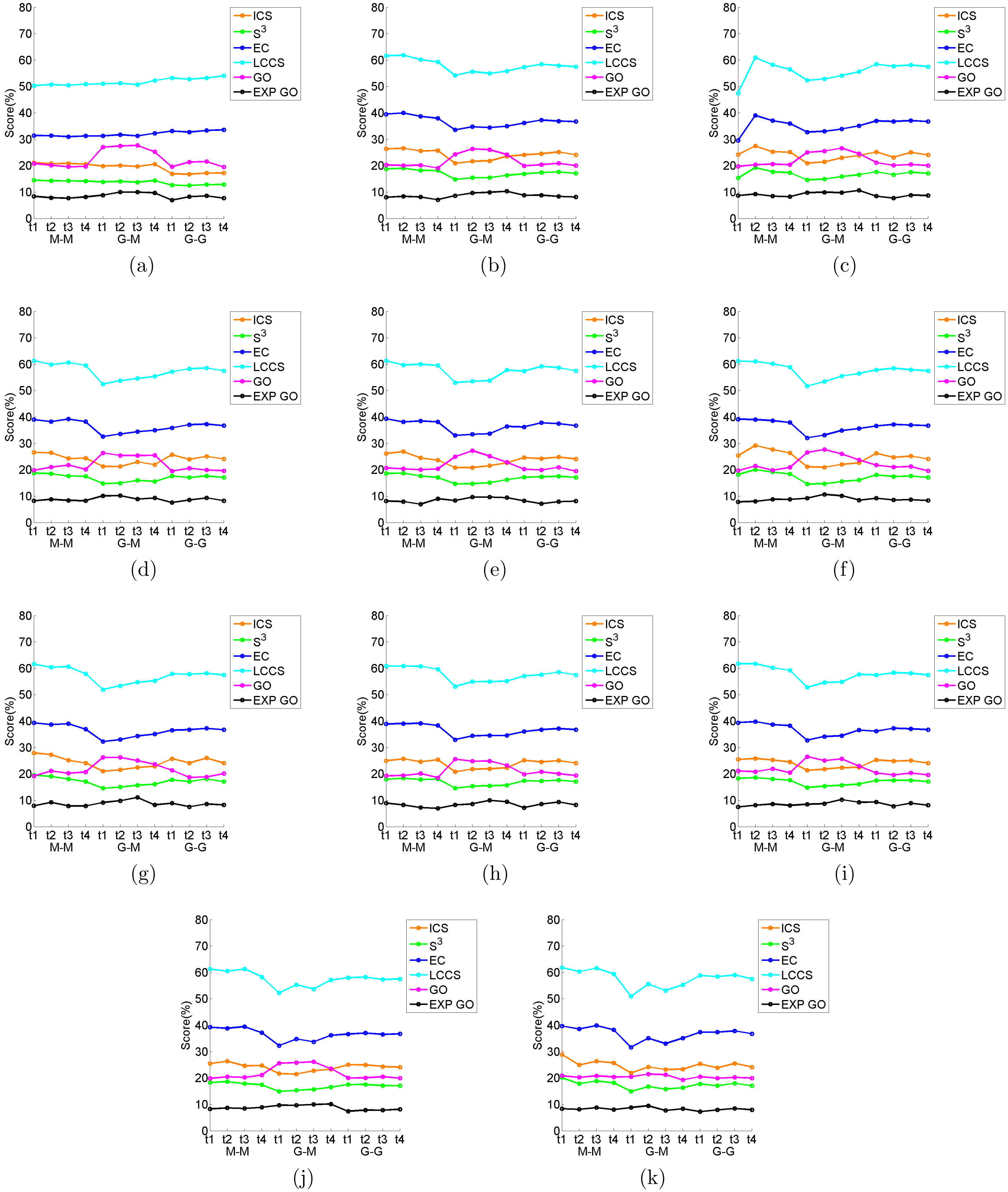

Supplementary Figure 23. Quality of Fly-Yeast alignments, with respect to the highest raw scores as a function of the aligner. Values of α by panel: **(a)** $\alpha = 0.0$, **(b)** $\alpha = 0.1$,**(c)** $\alpha = 0.2$,**(d)** $\alpha = 0.3$,**(e)** $\alpha = 0.4$,**(f)** $\alpha = 0.5$,**(g)** $\alpha = 0.6$,**(h)** $\alpha = 0.7$,**(i)** $\alpha = 0.8$,**(j)** $\alpha = 0.9$,**(k)** $\alpha = 1.0$.

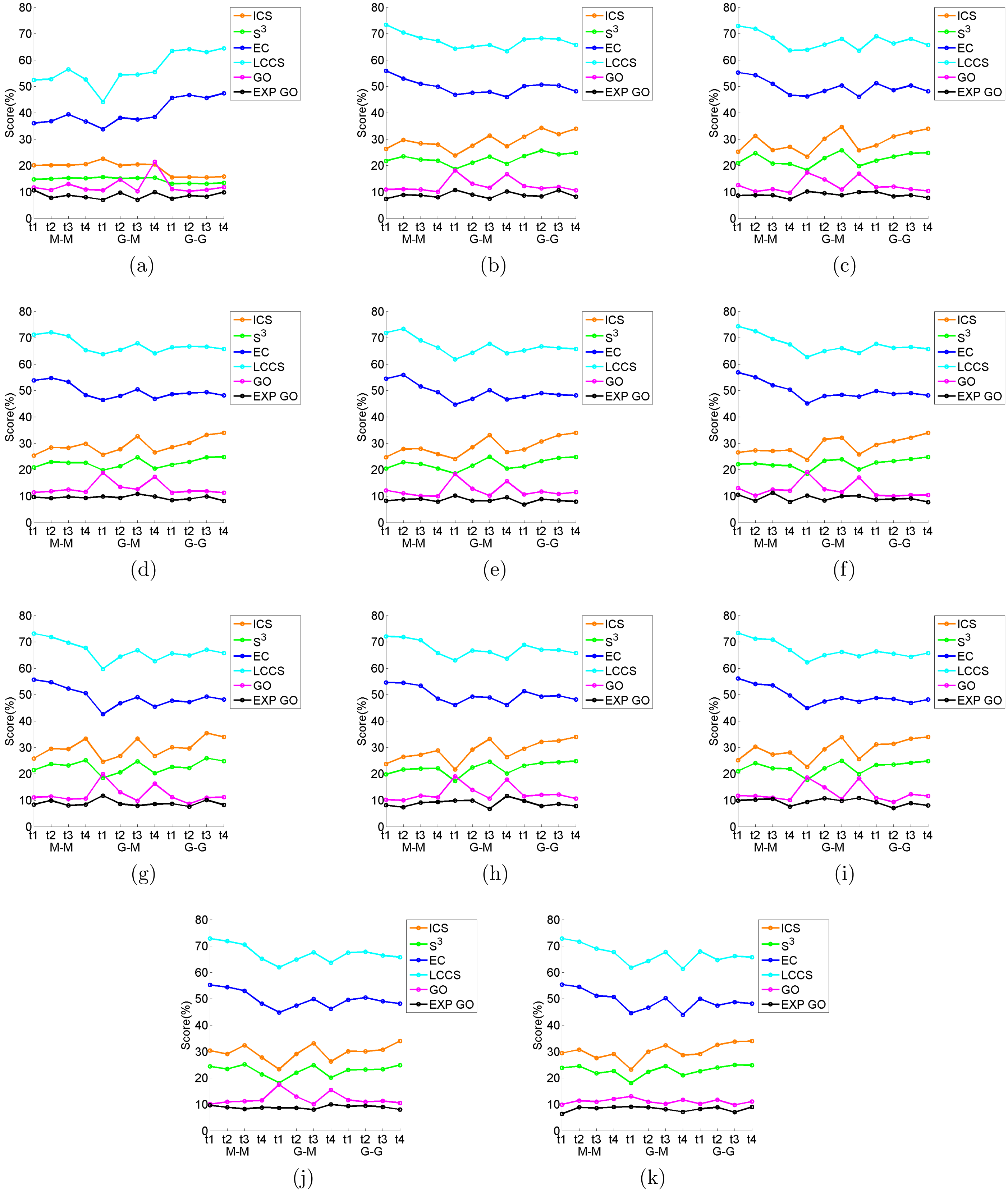

Supplementary Figure 24. Quality of Fly-Worm alignments, with respect to the highest <u>raw</u> scores as a function of the aligner. Values of α by panel: **(a)** $\alpha = 0.0$, **(b)** $\alpha = 0.1$,**(c)** $\alpha = 0.2$,**(d)** $\alpha = 0.3$,**(e)** $\alpha = 0.4$,**(f)** $\alpha = 0.5$,**(g)** $\alpha = 0.6$,**(h)** $\alpha = 0.7$,**(i)** $\alpha = 0.8$,**(j)** $\alpha = 0.9$,**(k)** $\alpha = 1.0$.

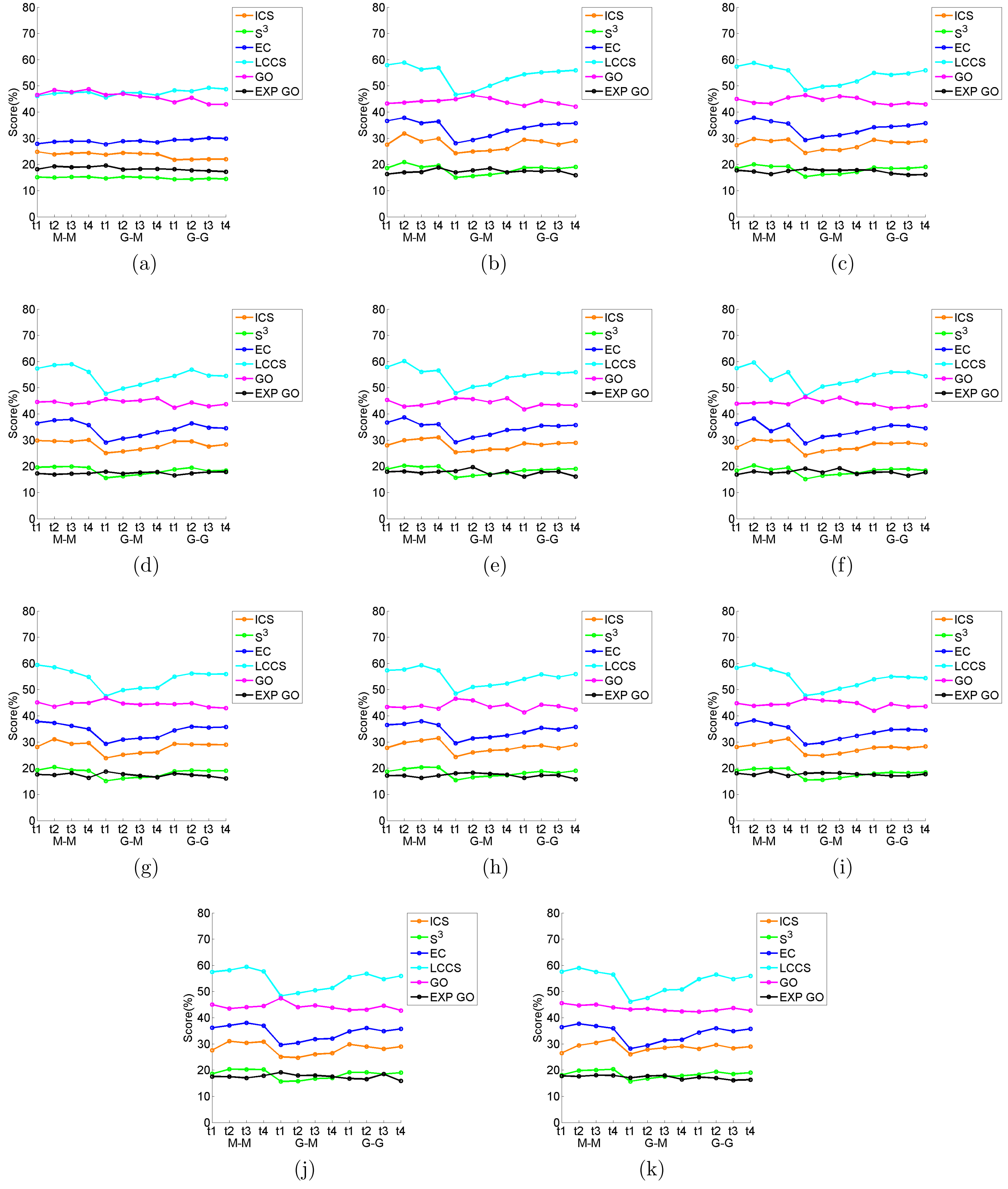

Supplementary Figure 25. Quality of Human-Yeast alignments, with respect to the highest raw scores as a function of the aligner. Values of α by panel: **(a)** $\alpha = 0.0$, **(b)** $\alpha = 0.1$,**(c)** $\alpha = 0.2$,**(d)** $\alpha = 0.3$,**(e)** $\alpha = 0.4$,**(f)** $\alpha = 0.5$,**(g)** $\alpha = 0.6$,**(h)** $\alpha = 0.7$,**(i)** $\alpha = 0.8$,**(j)** $\alpha = 0.9$,**(k)** $\alpha = 1.0$.

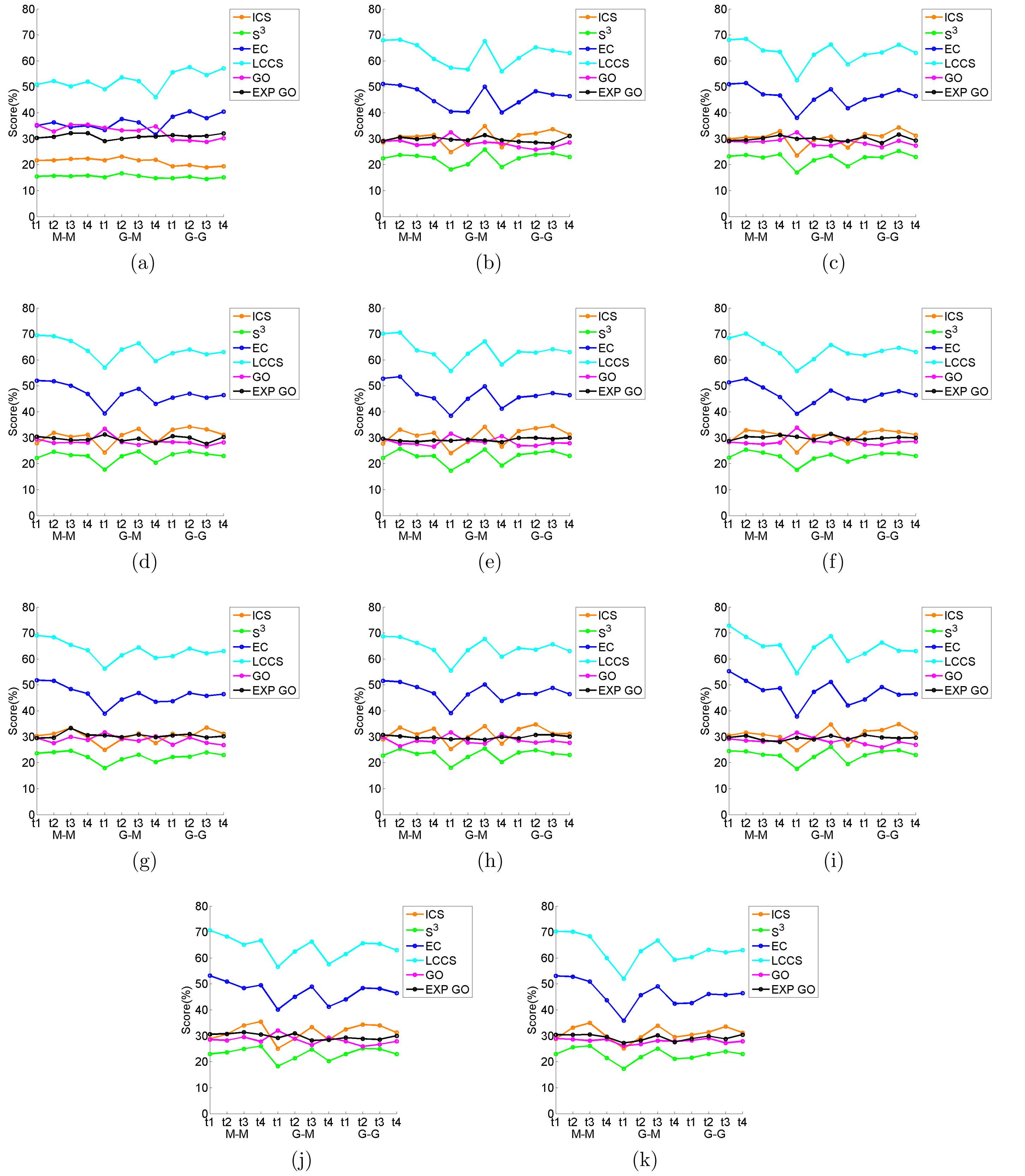

Supplementary Figure 26. Quality of Human-Worm alignments, with respect to the highest raw scores as a function of the aligner. Values of α by panel: **(a)** $\alpha = 0.0$, **(b)** $\alpha = 0.1$,**(c)** $\alpha = 0.2$,**(d)** $\alpha = 0.3$,**(e)** $\alpha = 0.4$,**(f)** $\alpha = 0.5$,**(g)** $\alpha = 0.6$,**(h)** $\alpha = 0.7$,**(i)** $\alpha = 0.8$,**(j)** $\alpha = 0.9$,**(k)** $\alpha = 1.0$.

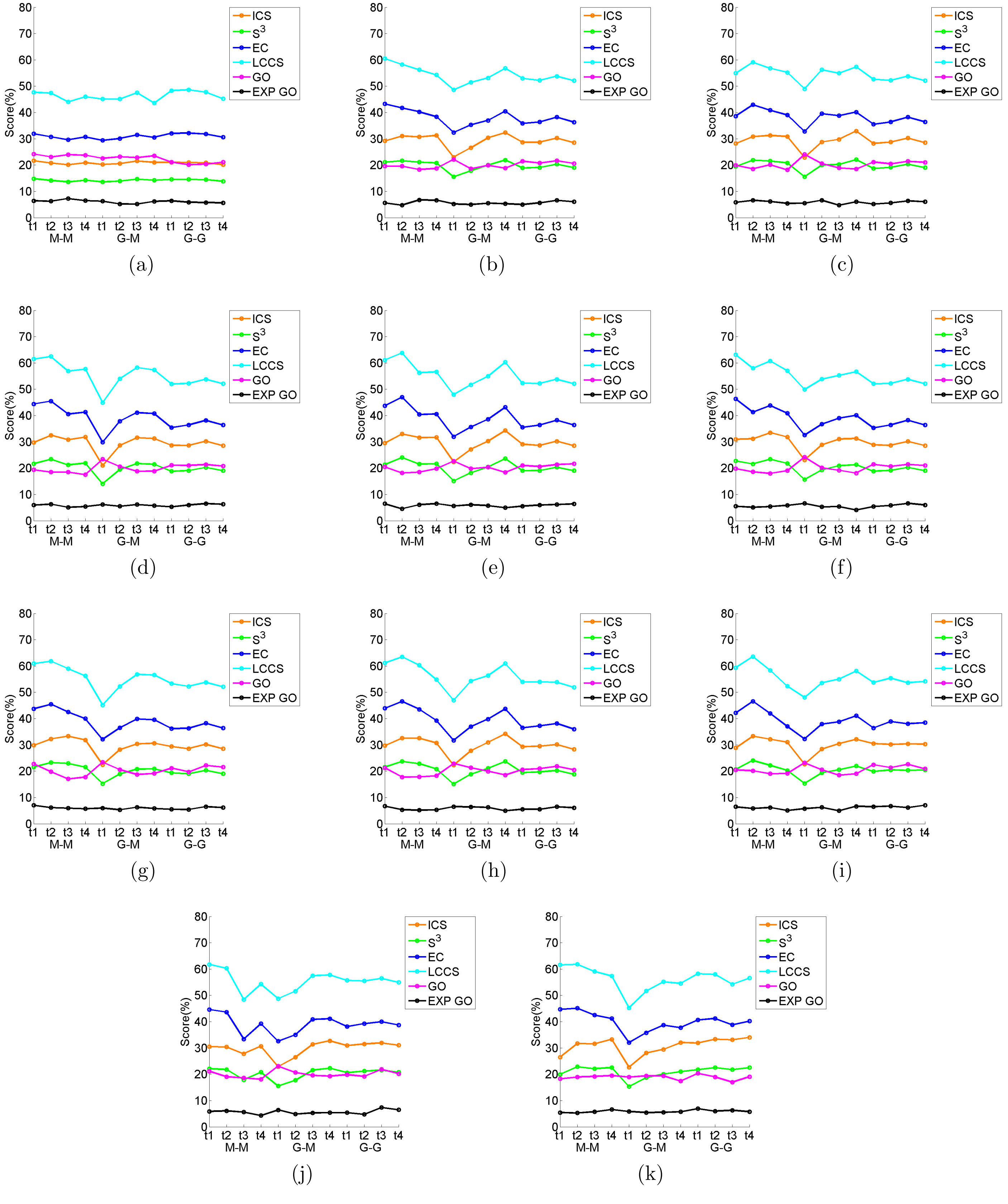

Supplementary Figure 27. Quality of Worm-Yeast alignments, with respect to the highest raw scores as a function of the aligner. Values of α by panel: **(a)** $\alpha = 0.0$, **(b)** $\alpha = 0.1$,**(c)** $\alpha = 0.2$,**(d)** $\alpha = 0.3$,**(e)** $\alpha = 0.4$,**(f)** $\alpha = 0.5$,**(g)** $\alpha = 0.6$,**(h)** $\alpha = 0.7$,**(i)** $\alpha = 0.8$,**(j)** $\alpha = 0.9$,**(k)** $\alpha = 1.0$.

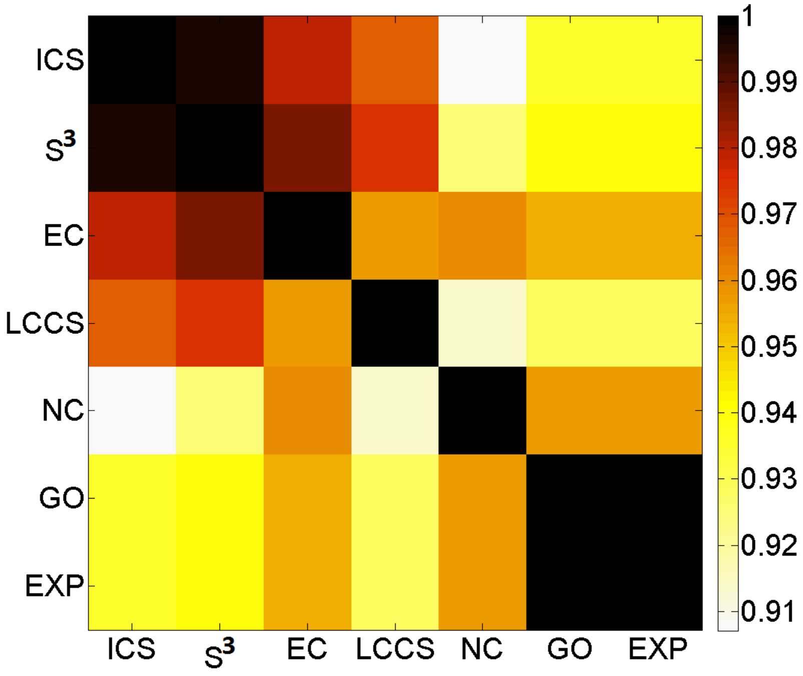

(a)

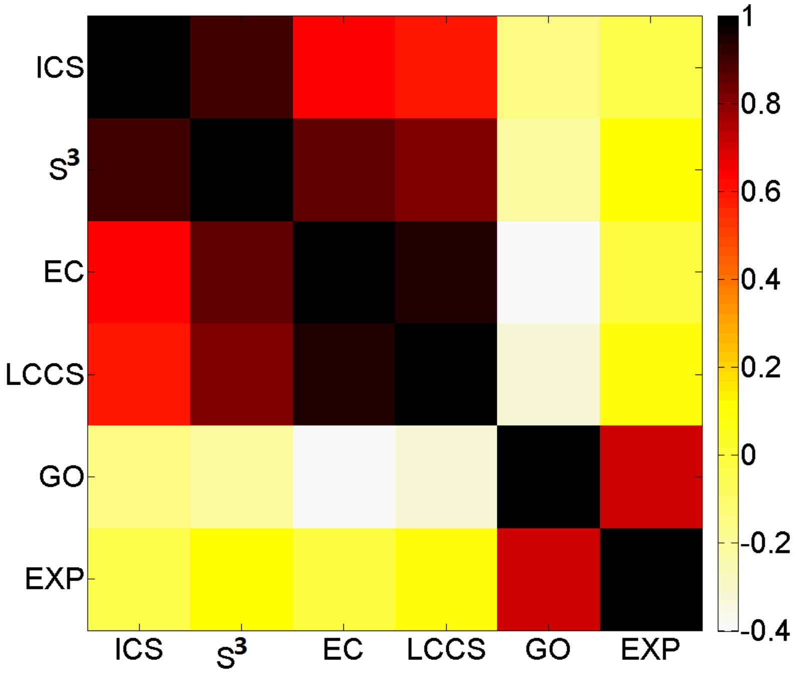

(b)

Supplementary Figure 28. Pairwise Spearman correlations between different alignment quality measures for: (a) synthetic networks with known ground truth node mapping and (b) real networks with unknown node mapping. Correlations were computed over alignments with the highest NC scores in panel (a) and over alignments with the highest EC scores in panel (b) (because we do not known NC scores for alignments of real networks). Note that color scales for the two panels are different.
